# Supplementary material for: Drug Repurposing: The Anthelmintics Niclosamide and Nitazoxanide Are Potent TMEM16A Antagonists That Fully Bronchodilate Airways
Source: Front Pharmacol. 2019 Feb 14;10:51. doi: 10.3389/fphar.2019.00051 (PMC6382696; doi:10.3389/fphar.2019.00051)
Supplement: Supplementary file 1 [file Data_Sheet_1.pdf]

Supplementary Material

Drug Repurposing: The Anthelmintics Niclosamide and Nitazoxanide are Potent TMEM16A Antagonists that Fully Bronchodilate Airways

Kent Miner[1], Katja Labitzke[2,†], Benxian Liu[1], Paul Wang[3], Kathryn Henckels[1], Kevin Gaida[1], Robin Elliott[1], Jian Jeffrey Chen[4], Longbin Liu[4,†], Anh Leith[5,†], Esther Trueblood[6,7,8†], Kelly Hensley[6,7,8†], Xing-Zhong Xia[1], Oliver Homann[9], Brian Bennett[1], Mike Fiorino[1], John Whoriskey[1], Gang Yu[1], Sabine Escobar[5], Min Wong[1], Teresa L. Born[5,†], Alison Budelsky[5,†], Mike Comeau[5], Dirk Smith[5,†], Jonathan Phillips[1], James A. Johnston[1], Joe McGivern[3], Kerstin Weikl[2,†], David Powers[3], Karl Kunzelmann[10], Deanna Mohn[1,*], Andreas Hochheimer[2,†,*], John K. Sullivan[1,†,*]

*** Correspondence:** Corresponding Authors: jksullivan@polestarbio.com (J.K.S); andreas.hochheimer@isarbioscience.de (A.H.); dmohn@amgen.com (D.M.)

Supplementary Data

1 Supplementary Methods

1.1 Measuring antagonist effects on bronchial smooth muscle cell (BSMC) pro-contractile calcium flux.

Human BSMCs were obtained from Lonza, resuspended, cultured and serum starved for 24 hours as described for the histamine membrane potential assay. Compounds and dilutions were prepared in same manner, except they were serially diluted in Assay Buffer (2 mM CaCl2, 1 mM MgCl2, 4.5 mM KCl, 155 mM NaCl, 5 mM HEPES, 10 mM glucose, pH7.4) to 4X the final concentration. A Calcium Indicator Working Solution (10 ml) was prepared by adding 1ml BD PBX Signal Enhancer (Cat# 51-9006254) and 10 µl of BD Calcium Indicator (BD Cat# 850000 resuspended in 100% DMSO) to 9 ml Assay Buffer. Following serum starvation of the BSMCs, culture media was aspirated off and 50µl of Calcium Indicator Working Solution was added to each well of microtiter plate. Twenty-five microliters of the 4X serially diluted compounds were then added and cells were incubated with compounds for 30 minutes at room temperature. Twenty-five microliters of a 4X histamine or methacholine solution (where 1X reflects EC_{80}-EC_{100} concentration from earlier validation of aliquot of cells from same donor) was then added and fluorescence changes were measured by a FLIPR-Tetra or internal FlashFluor instrument. In some experiments the TMEM16A opener, Eact, was used in place of histamine or methacholine.

1.2 Effects of the TMEM16A opener Eact on leukotriene secretion and Muc5AC mucin production from human bronchial epithelial air-liquid interface cultures.

Normal, asthma and COPD human bronchial epithelial (HBE) cells were from Lonza (Walkersville, MD). Cells were grown on 0.4 μM transparent PET transwell membranes insert (BD biosciences, San Diego). S-ALI air-liquid interface media (Lonza) was changed every other day until the cells reached confluence, at which time the apical medium was removed to establish an air liquid interface cultures. The basolateral medium was changed every other day. All experimentation was carried out on day 14-18 after ALI establishment. At this point, mature secretory cells are present in these differentiating cultures and the cells respond with maximal proliferation to IL-13 (10 ng/ml) (Peprotech, Rocky Hill, NJ, USA).

Human ALI cultures from a normal donor were treated with Eact, Eact vehicle and 10 ng/ml IL13 in basolateral medium for 48 hrs. After 48hr, 100 μl of basolateral medium were collected for determining leukotriene B4 and C4 secretion using ELISA kits from Enzo Life Sciences, Inc. and LSBio LifeSpan Biosciences Inc., respectively, and by following manufacture's protocols. There was a n of 3 replicate ALI cultures per condition.

The determine the effects of Eact on mucin secretion, mature human bronchial epithelial ALI cultures established from normal, asthma and COPD patients (2 donors, each) were treated with vehicle or 5 μM Eact in basal media for 72 hours. To collect mucus, the apical side of ALI cultures (n = 2 ALIs per donor) was washed two times with 0.5 ml of 10 mM DTT (37°C for 15 min, each) and the washes were combined. Levels of Muc5AC protein in apical washes was then determined using a Muc5AC ELISA kit (Cosmo Bio USA, Carlsbad, CA) and following the manufacture's protocol.

2 Supplementary Tables

2.1 Supplementary Table 1. High-throughput-screening for TMEM16A antagonists and hit triaging.

HTS Campaign Activity	Small molecule hits	Cell line	Assay modality	Test cpd. incubation/ stimulation	Hit cut-off	S/B	Avg. RZ/RZ'
**Small molecule library of 581,849 compounds**							
TMEM16A Primary Screen (n=1, 5μM final)	1,445	HEK293T:eYFP:TMEM16A(abc)	FI eYFP	30 min @ RT / 10mM Iodide, 2μM Ionomycin (final)	POC <72.6%	6.5	0.83 / 0.83
Hit Confirmation (n=3, 5μM final)	673	HEK293T:eYFP:TMEM16A(abc)	FI eYFP	same as above	POC <72.6%	6.5	0.76 / 0.80
Validation to remove compounds showing autofluorescence (n=1, 5μM final)	491	HEK293T:eYFP:TMEM16A(abc)	FI	30 min @ RT	Baseline FI <10x STDV of neutral control	n.a.	n.a.
Validate compounds not affecting calcium flux or $[Ca^{2+}]_i$ (dose-response, 22-step, 1:2 dilution)	328	HEK293T	FI Calcium 6	45 min @ RT / 1μM Ionomycin (final)	IC_{50} >10μM	29.5	0.74 / 0.81
TMEM16A Potency (dose response, 22-step, 1:2 dilution)	145	HEK293T:eYFP:TMEM16A(abc)	FI	30 min @ RT / 10mM Iodide, 2μM Ionomycin (final)	IC_{50} <5μM	6.2	0.76 / 0.79
Compound Quality Control	130	n.a.	Mass spec.	n.a.	≥50% purity, correct mass	n.a.	n.a.

High-throughput-screening of a small molecule library for TMEM16A antagonists using the halide-sensitive YFP (eYFP) assay was conducted in a fully automated and scheduled setup. The eYFP assay allows identification of TMEM16A inhibitors by measuring TMEM16A-dependent quenching of eYFP fluorescence intensity (FI). Cells were incubated with the test compound before the assay was started by addition of trigger solution containing ionomycin and iodide ions. Ionomycin triggers the Ca^{2+}-dependent activation of TMEM16A, which allows iodide ions to enter the cell and quench eYFP fluorescence intensity. Assay results were normalized based on the benchmark TMEM16A inhibitor benzbromarone (100% inhibition corresponding to 0% TMEM16A activity) and neutral control (0% inhibition corresponding to 100% TMEM16A activity) and reported as percent-of-control (POC). Hit cut-off is a statistical value that was calculated based on the results of all test wells (see Supplementary Figure 1). Compounds providing >27.4% inhibition corresponding to <72.6% POC TMEM16A activity were called hits. Statistical quality control parameters such RZ-factor and RZ'-factor and signal/background ratio (S/B) were determined throughout the HTS campaign. During primary screening the entire library was tested at a single concentration and yielded 1,445 primary hits. Confirmation screening tested all primary hits again in three independent, consecutive runs and delivered 673 confirmed hits. Auto-fluorescent compounds were eliminated based on the baseline fluorescence intensity recorded prior to addition of trigger solution and resulted in 491 non-fluorescent hits. These hits were next tested in dose response assays at 22 concentrations in calcium flux assays. This eliminated false-positive TMEM16A antagonists that inhibited ionomycin-dependent increase of intracellular calcium concentration. Dose response testing of the remaining hits revealed 145 hits with an IC_{50}<5μM and finally 130 validated hits, which passed quality control by mass spec analysis. Further details are provided in the materials and methods section.

2.2 Supplementary Table 2. Patient Demographics (Cultured Human Bronchial Smooth Muscle Cells Used in RNA-Seq)

Gender (Race)	Age (Years)	Cause of Death	Medical History - Respiratory *Disease (Duration), Additional Comments*	Medications (Respiratory)	Smoking History? (Yrs.; Packs/Day)
Male (C)	11	Accidental	None	None	No
Male (C)	60	Anoxic Event	None	None	No
Male (B)	4	Accidental	Asthma (unknown)	Albuterol	No
Female (H)	19	Anoxia/asthma	Asthma (17.5 yrs.), 2 prior admittances for severe asthma before death	None Specified	No
Male (C)	27	Accidental	Asthma (20 yrs.)	Proventil, Albuterol	No
Male (C)	44	Anoxic Event	COPD (unknown)	Advair, Combivent	Yes (25; 0.5)
Female (C)	48	Anoxic Event	COPD (unknown), diagnosed 6 months before death with emphysema	None Specified	Yes (34; 2-3)
Male (C)	49	Anoxic Event	COPD (unknown)	None Specified	Yes (34; 2-3)

Cells were from Lonza Walkersville Inc., Walkersville, MD, USA. Bronchial cells were acquired from deceased donors and isolated from the bottom of the trachea and the bifurcation of the bronchi. Medical history was obtained from hospital records. Smoking refers to cigarettes, unless otherwise specified. Abbreviations are as follows: C, Caucasian; B, African-American; H, Hispanic. The RNA-Seq dataset containing primary human airway smooth muscle cells and bronchial epithelial cells has been described earlier (Aisenberg et al. 2016).

2.3 Supplementary Table 3. Patient Demographics (Cultured Human Bronchial Epithelial Cells Used in RNA-Seq)

Gender (Race)	Age (Years)	Cause of Death	Medical History - Respiratory *Disease (Duration), Additional Comments*	Medications (Respiratory)	Smoking History? (Yrs.; Packs/Day)
Male (C)	11	Accidental	None	None	No
Female (B)	17	Anoxic Event	None	None	No
Male (C)	32	Drug Overdose	None	None	No
Male (C)	39	Drug Overdose	None	None	Yes (occasional)
Male (C)	46	Anoxic Event	None	None	Yes (30; 2)
Male (C)	47	Anoxic Event	None	None	Yes (30; 3 cigars/d)
Male (B)	54	Anoxic Event	None	None	Yes (30; 1)
Female (H)	19	Anoxia, Asthma	Asthma (17.5 yrs.), 2 prior admittances for severe asthma attacks prior to death	None Specified	No
Male (C)	52	Anoxic Event	Asthma (diagnosed as child), bronchitis x 3 months prior to death	Albuterol, Advair, Singulair, Prednisone, Decadron	No
Male (H)	57	Anoxia, Asthma	Asthma (unknown), admitted 1 day prior to death for asthmatic event	Inhaler, Prednisone, Albuterol	Yes (?, 1-2 cigs/d)
Female (B)	63	Anoxia, Asthma	Asthma (30+ yrs.)	Daily inhaled steroid	No
Male (C)	67	Anoxic Event	Asthma (10 yrs.)	None Specified	No
Male (C)	40	Drug Overdose	COPD (unknown)	None Specified	Yes (25, 1)
Male (C)	55	Drug Overdose	COPD (unknown)	None Specified	Yes (40, 1-2)
Female (C)	72	Anoxic Event	COPD (20 yrs.)	Prednisone	Yes (56, 2)

Cells were from Lonza Walkersville Inc., Walkersville, MD, USA. Bronchial cells were acquired from deceased donors and isolated from the bottom of the trachea and the bifurcation of the bronchi. Medical history was obtained from hospital records. Smoking refers to cigarettes, unless otherwise specified. Abbreviations are as follows: C, Caucasian; B, African-American; H, Hispanic; cigs, cigarettes. The RNA-Seq dataset containing primary human airway smooth muscle cells and bronchial epithelial cells has been described earlier (Aisenberg et al. 2016).

2.4 Supplementary Table 4. Patient Demographics (Human Bronchial Epithelial Cells Used in ALI Cultures and RNA-Seq)

Gender (Race)	Age (Years)	Cause of Death	Medical History - Respiratory *Disease (Duration), Additional Comments*	Medications (Respiratory)	Smoking History? (Yrs.; Packs/Day)
Male (C)	64	Accidental	None	None	Yes (unknown)
Female (H)	40	Anoxic Event – CV Stroke	None	None	No
Male (C)	56	Unspecified	COPD (unknown), hospitalized one month prior to death for emphysema	None Specified	Yes (25; 1)
Male (B)	52	Unspecified	COPD (unknown)	None Specified	Yes (12; 1)
Female (B)	52	Anoxic Event	COPD (unknown)	Inhaler	Yes (25; 1)

Cells were from Lonza Walkersville Inc., Walkersville, MD, USA. Bronchial cells were acquired from deceased donors and isolated from the bottom of the trachea and the bifurcation of the bronchi. Medical history was obtained from hospital records. Smoking refers to cigarettes, unless otherwise specified. Abbreviations are as follows: C, Caucasian; B, African-American; H, Hispanic. The RNA-Seq dataset containing primary human airway smooth muscle cells and bronchial epithelial cells has been described earlier (Aisenberg et al. 2016).

3 Supplementary Figures

3.1 Supplementary Figure 1

A RZ'-factor and signal distribution per plate over entire HTS campaign

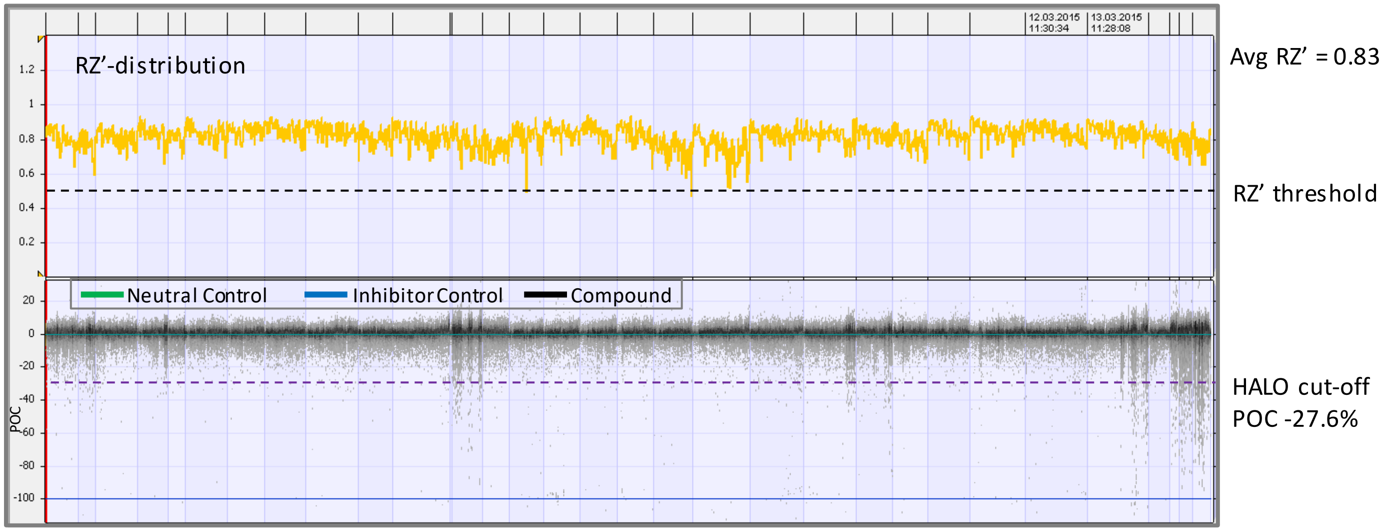

B Average HTS quality control metrics

Avg	S/B	Z' Factor	RZ' Factor	Z Factor	RZ Factor
HTS	6.5	0.76	0.83	0.74	0.83

Supplementary Figure 1. Signal distribution and quality control metrics of the TMEM16A HTS campaign. Data analysis of the entire HTS campaign was performed using Genedata Screener. **(A)** RZ'-factor and signal distribution per plate over entire HTS campaign. RZ'-factor was calculated per plate and is plotted per plate over the entire HTS campaign (top panel). Average RZ' was 0.83 and RZ' threshold was set at 0.5. Normalization of library compound activities was based on neutral control (0% inhibition) and on TMEM16A inhibitor control Benzbromarone (-100% inhibition) are are plotted as percent-of-control (POC)(bottom panel). Unbiased hit threshold is given as HALO (Hit Assessment and Lead Optimization) cut-off, which was calculated as Median+3xIQR+20% of all test wells. **(B)** Average HTS quality control metrics. Statistical quality control parameters such Z-factor, Z'-factor, RZ-factor, RZ'-factor and signal/background ratio (S/B) were determined throughout the HTS campaign and are summarized as average values.

3.2 Supplementary Figure 2

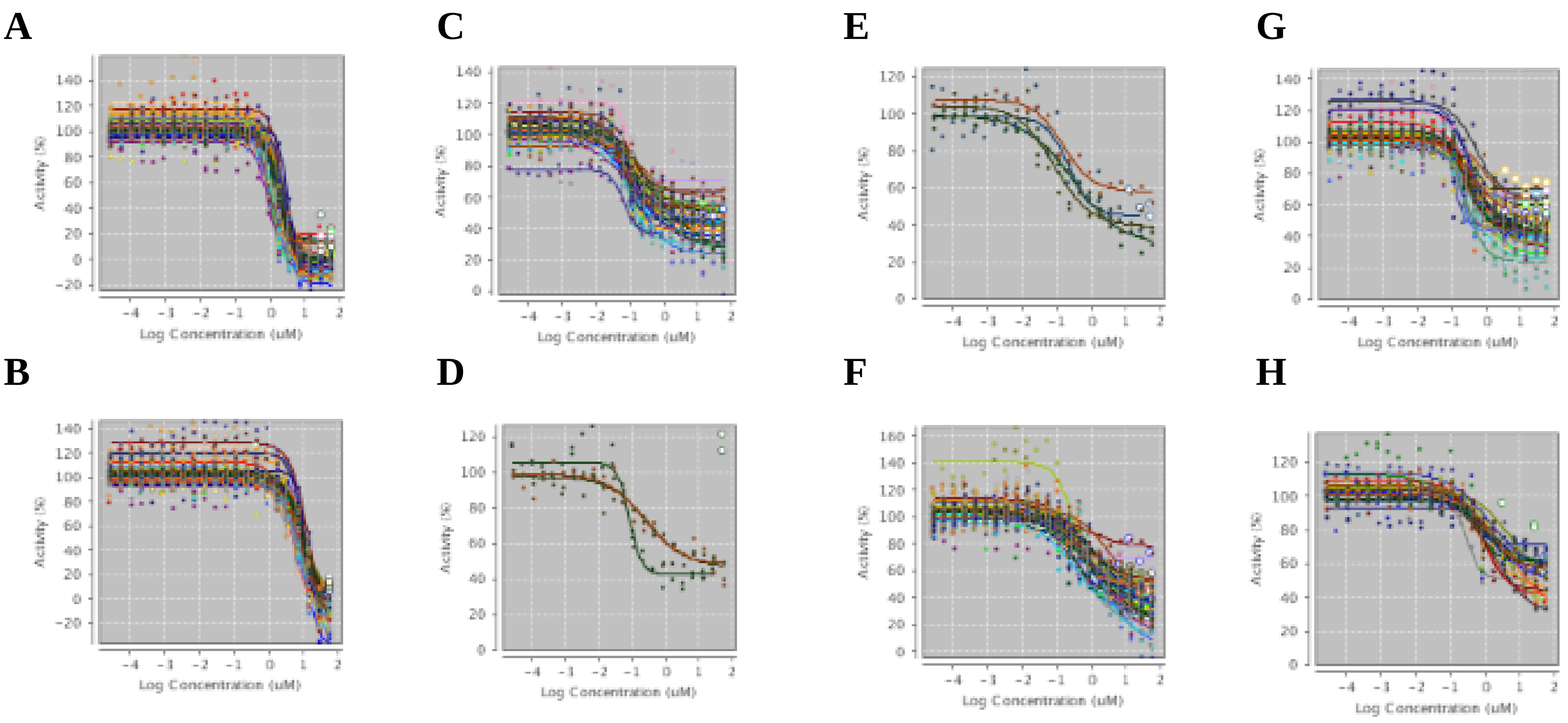

Supplementary Figure 2. **Composite dose-response data of compound impact in blocking ionomycin-induced TMEM16A (abc) eYFP quenching, where iodide was used as the permeant anion.** Shown are the results of separate experiments run on differing days. Benzbromarone **(A)** and CaCCinh-A01 **(B)** fully blocked the iodide eYFP response, with IC_{50} (µM) of 1.5081 ± .504 (n=58) and 8.125 ± 2.72 (n=58), while niclosamide **(C)**, Cpds 2-5 **(D-G)** and the benchmark TMEM16A antagonist 1PBC **(H)** showed potent but partial block of the ionomycin-induced eYFP response with IC_{50} (µM) values of 0.13208 ± 0.0671 (n=42), 0.19643 ± 0.105 (n=3), 0.184 ± 0.0891 (n=4), 0.95369 ± 0.648 (n=54), 0.36967 ± 0.131 (n=42) and 1.5233 ± 1.36 (n=17), respectively. Activity (%) is the same as Percent of Control (POC), where 100% is no block of the ionomycin-induced iodide quenching of the TMEM16A eYFP response, while 0% is full inhibition of the TMEM16A halide sensitive YFP response.

3.3 Supplementary Figure 3

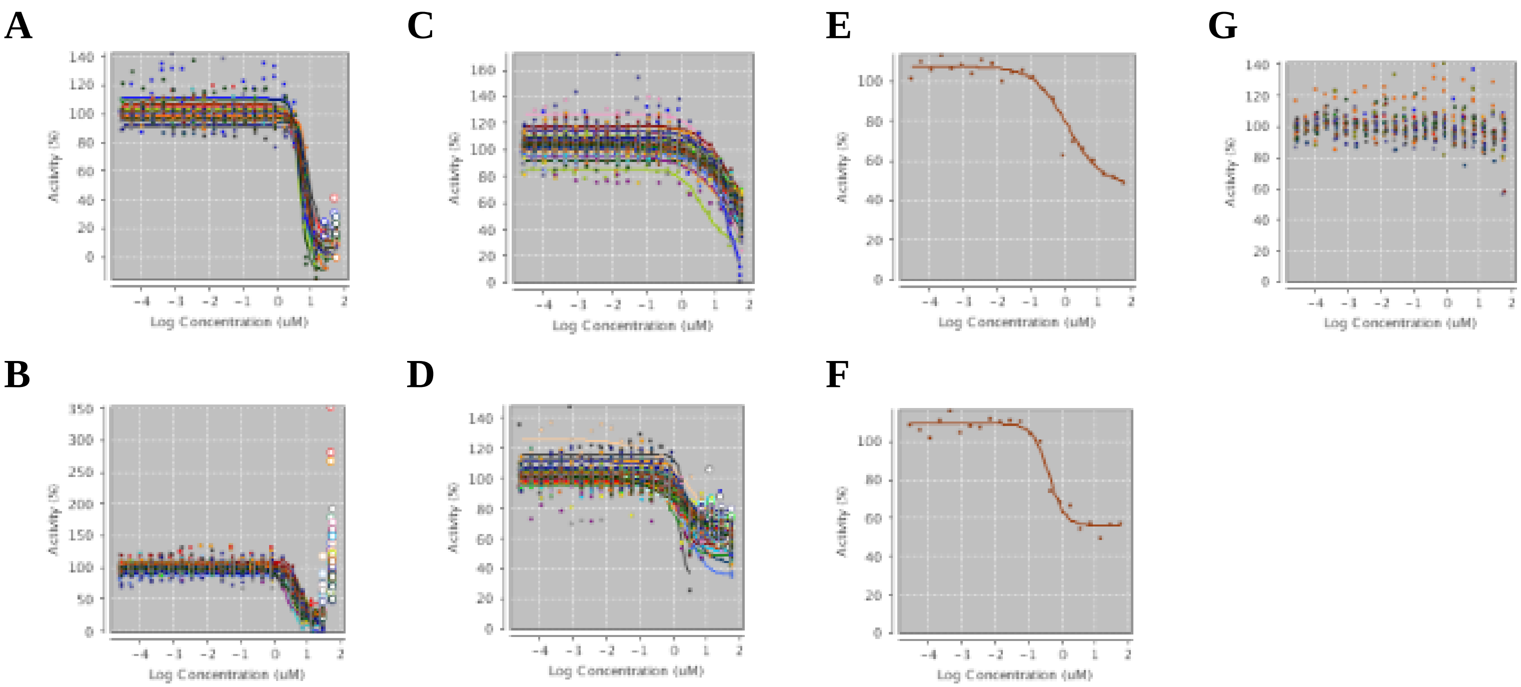

Supplementary Figure 3. **Composite dose-response data of compound impact in blocking ionomycin-induced TMEM16A (abc) eYFP quenching, where iodide was used as the permeant anion.** Shown are the results of separate experiments run on differing days with maximum concentration being 55.6 µM. MONNA **(A)** and dichlorophen **(B)** fully blocked the iodide eYFP response, with IC_{50} (µM) of 6.2947 ± 1.47 (n=19) and 4.3647 ± 1.21 (n=58), while niflumic acid **(C)** showed weak activity and IC_{50} (µM) of 26.57 ± 11.8 (n=55). The benchmark antagonist NTTP **(D)**, the approved drug nitazoxanide **(E)** and its metabolic product tizoxanide **(F)** showed potent, but partial inhibition with IC_{50} values (µM) of 2.476 ± 1.236 (n=46), 1.23 (n=1) and 0.398 (n=1), respectively. The benchmark TMEM16A antagonist, T16Ainh-A01 **(G)**, was inactive (IC_{50} > 55.6 µM, n= 16) in blocking the ionomycin-induced eYFP response. We were also unable to show any significant activity of this compound by QPatch electrophysiology using HEK293/TMEM16A (acd) cells and elevated intracellular calcium (Table 1 and Supplementary Figure 9), while all the other compounds were confirmed active in this ephys assay. The loss of dichlorophen activity at the highest doses is most likely due to poor compound solubility at higher concentrations. Activity (%) is the same as Percent of Control (POC), where 100% is no block of the ionomycin-induced iodide quenching of the TMEM16A eYFP response, while 0% is full inhibition of the TMEM16A halide sensitive YFP response.

3.4 Supplementary Figure 4

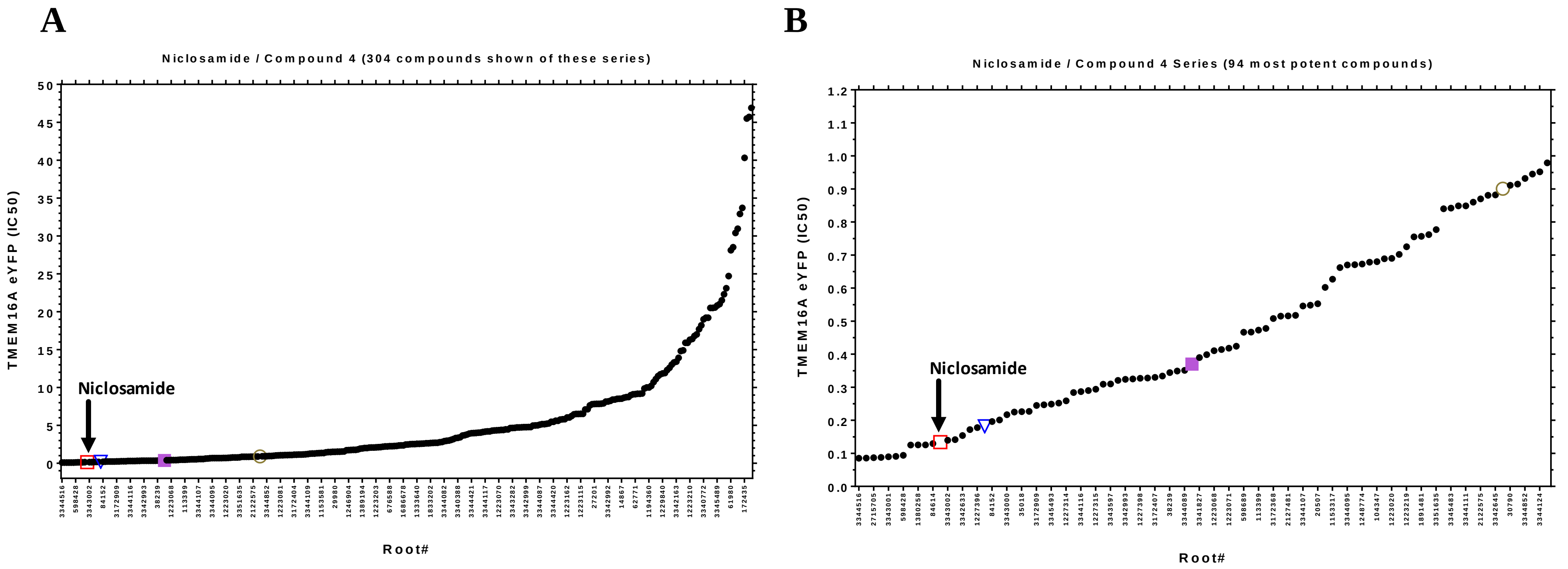

Supplementary Figure 4. **Distribution of potency of Niclosamide related compounds in the TMEM16A halide-sensitive YFP assay.** A total of 304 distinct compounds from the niclosamide and Cpd 4 series had IC_{50} < 50 µM **(A)**, with the 94 most potent compounds provided in **(B)**. An additional 78 compounds aren't displayed as they were considered inactive having an IC_{50} > 50 µM. The compound identification number (root#) is listed on x-axis versus the potency (IC_{50}, µM) on y-axis. It should be noted due to the large number of compounds shown, the x-axis only provides a subset of the root identification numbers that are plotted. The location of niclosamide (Cpd 1) (red, open square), Cpd 2 (blue, open triangle), Cpd 3 (84152 label on x-axis), Cpd 4 (brown, open circle) and Cpd 5 (purple, solid square) are shown in the plots.

3.5 Supplementary Figure 5

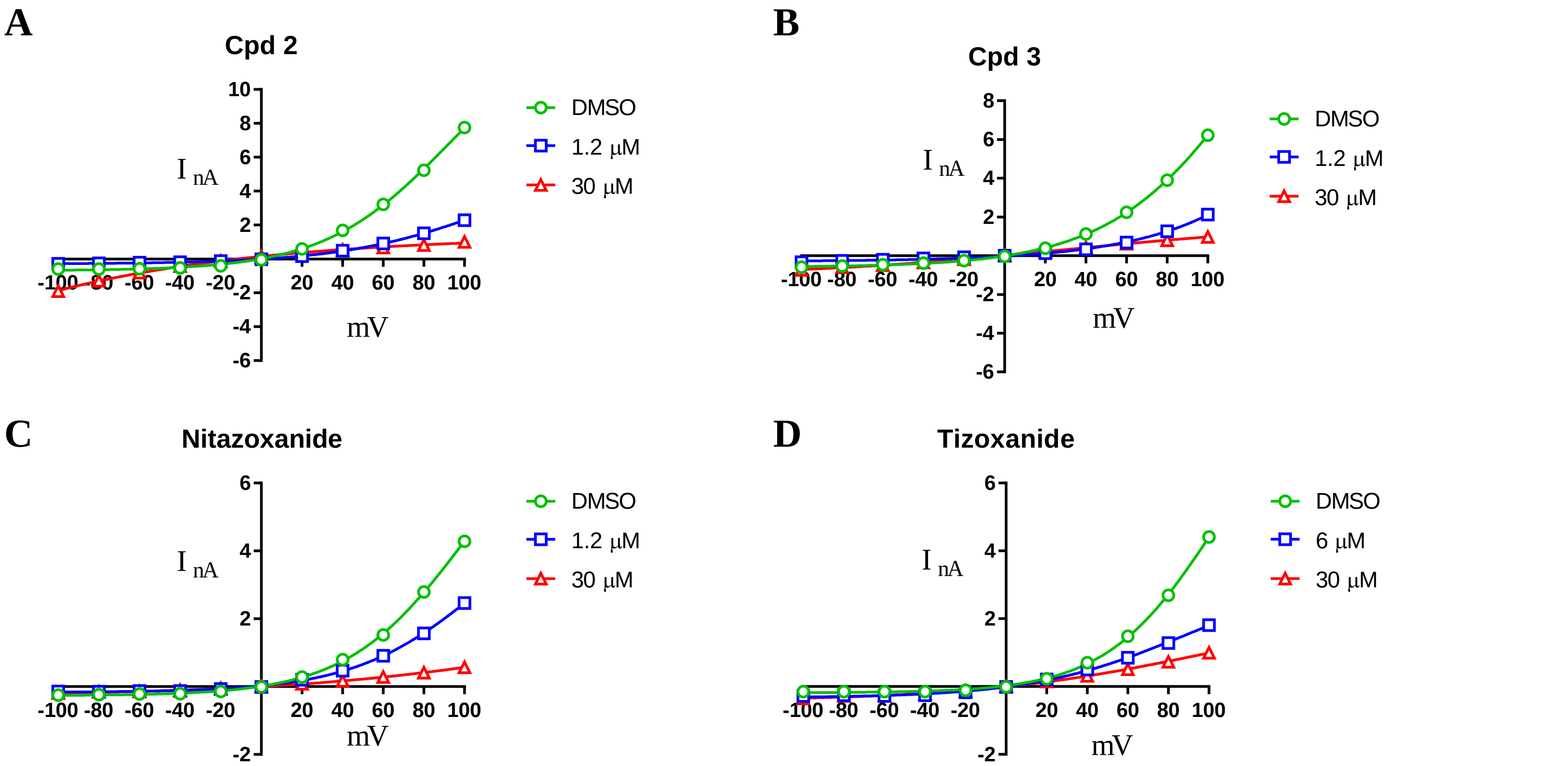

Supplementary Figure 5. **HEK293 / TMEM16A (acd) QPatch electrophysiology assay using 170 nM free $[Ca^{2+}]_i$.** Representative current/voltage (I/V) traces of Cpd 2, Cpd 3, Nitazoxanide and Tizoxanide are shown in panels **A-D**, respectively. The voltage protocol involved 20 mV steps from -100 to +100 mV from a holding potential of 0 mV. The currents were voltage-dependent and outward rectifying as expected for TMEM16A recordings in the presence of low intracellular calcium.

3.6 Supplementary Figure 6

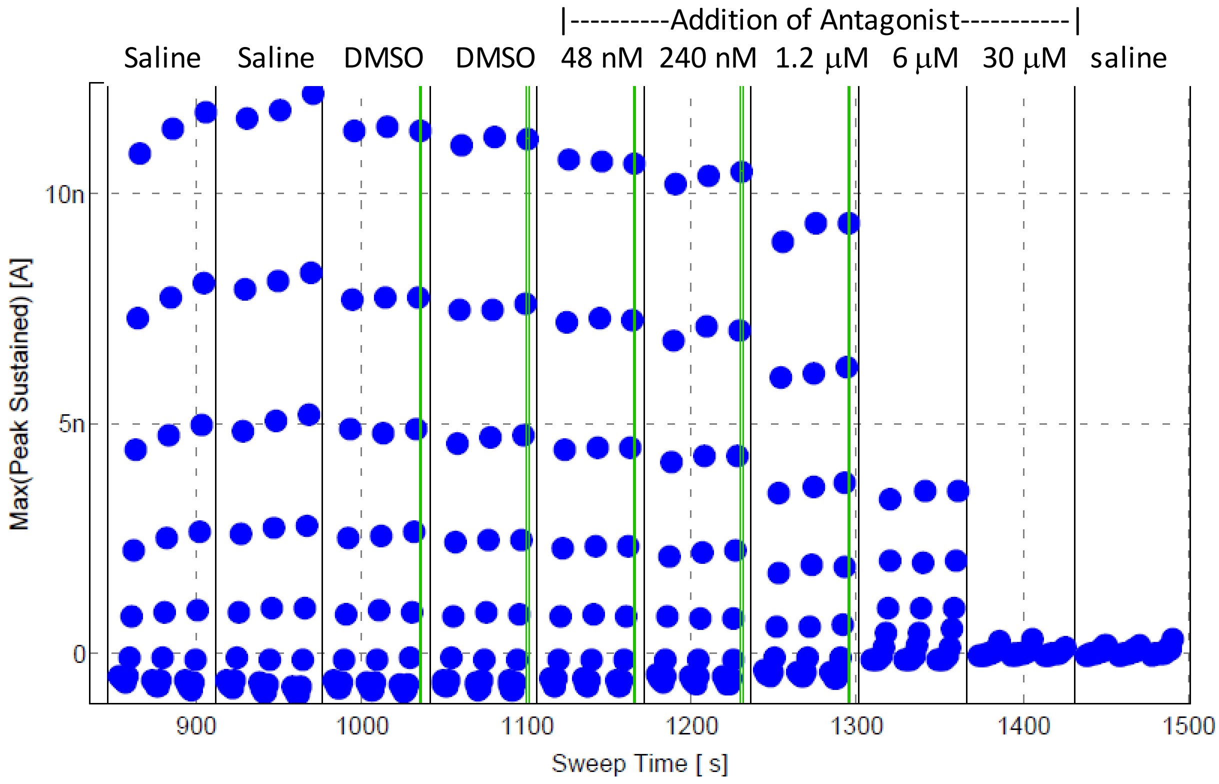

Supplementary Figure 6. **Impact of benzbromarone on TMEM16A (acd) calcium-activated chloride currents as measured by QPatch electrophysiology.** Shown is a representative recording of the impact of progressive (5-fold) increases of benzbromarone on the TMEM16A current-voltage response. Three I/V protocols were run from -100 to +100 mV (20 mV steps) from a Vhold of 0 mV for each sample addition, which allowed at least 60s incubation time per compound dose in our standard QPatch assay for determining potency (see Materials and Methods). The dots reflect the measured current (nA) after each 20 mV step from -100 to +100 mV. The currents were voltage-dependent in presence of 170 nM free intracellular calcium and benzbromarone inhibited both the outward and inward currents.

3.7 Supplementary Figure 7

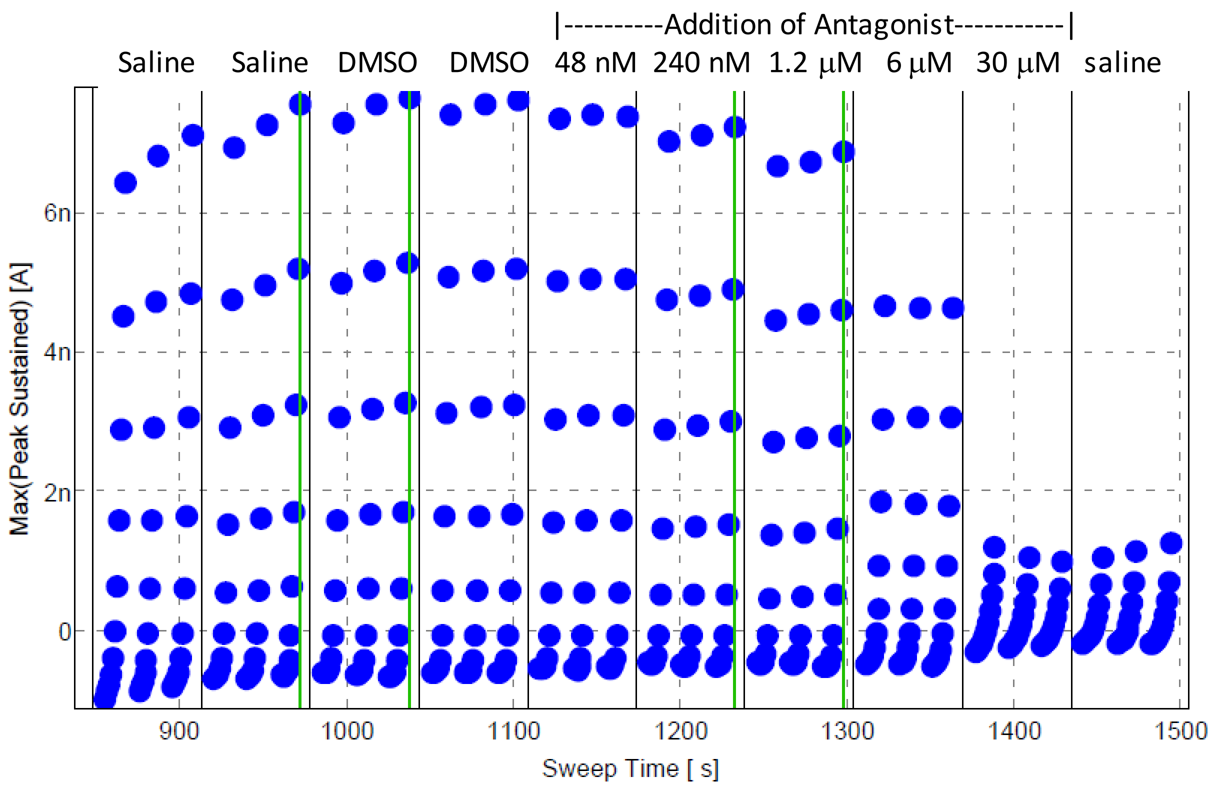

Supplementary Figure 7. Impact of CaCCinh-A01 on TMEM16A (acd) calcium-activated chloride currents as measured by QPatch electrophysiology. Shown is a representative recording of the impact of progressive (5-fold) increases of CaCCinh-A01 on the TMEM16A current-voltage response. Three I/V protocols were run from -100 to +100 mV (20 mV steps) from a Vhold of 0 mV for each sample addition, which allowed at least 60s incubation time per compound dose in our standard QPatch assay for determining potency (see Materials and Methods). The channel exhibited the expected voltage-dependence with these recordings using 170 nM free intracellular calcium and CaCCinh-A01 inhibited both the outward and inward currents.

3.8 Supplementary Figure 8

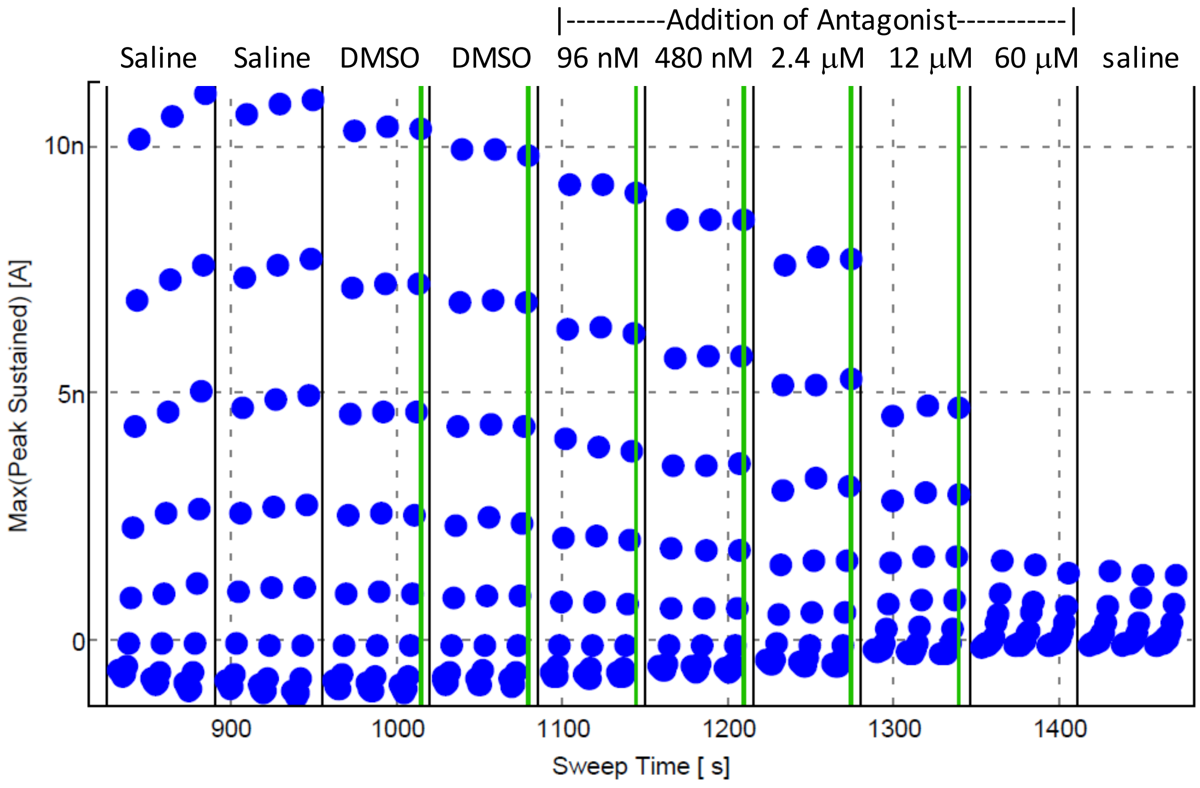

Supplementary Figure 8. **Impact of MONNA on TMEM16A (acd) calcium-activated chloride currents as measured by QPatch electrophysiology.** Shown is a representative recording of the impact of progressive (5-fold) increases of MONNA on the TMEM16A current-voltage response. Three I/V protocols were run from -100 to +100 mV (20 mV steps) from a Vhold of 0 mV for each sample addition, which allowed at least 60s incubation time per compound dose in our standard QPatch assay for determining potency (see Materials and Methods). The channel exhibited the expected voltage-dependence with these recordings using 170 nM free intracellular calcium and MONNA inhibited both the outward and inward currents.

3.9 Supplementary Figure 9

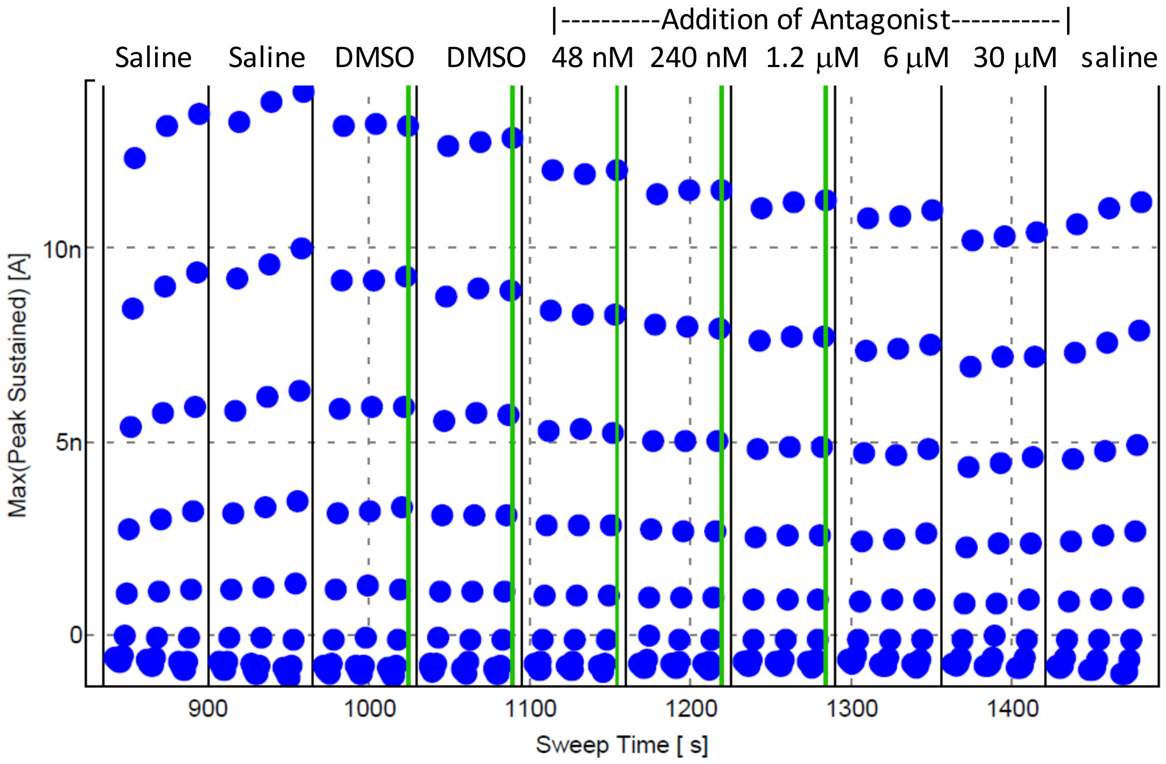

Supplementary Figure 9. **Impact of T16Ainh-A01 on TMEM16A (acd) calcium-activated chloride currents as measured by QPatch electrophysiology.** Shown is a representative recording of the impact of progressive (5-fold) increases of T16Ainh-A01 on the TMEM16A current-voltage response. Three I/V protocols were run from -100 to +100 mV (20 mV steps) from a Vhold of 0 mV for each sample addition, which allowed at least 60s incubation time per compound dose in our standard QPatch assay for determining potency (see Materials and Methods). The channel exhibited the expected voltage-dependence with these recordings using 170 nM free intracellular calcium and the compound T16Ainh-A01 showed only slight effects in blocking the TMEM16A currents.

3.10 Supplementary Figure 10

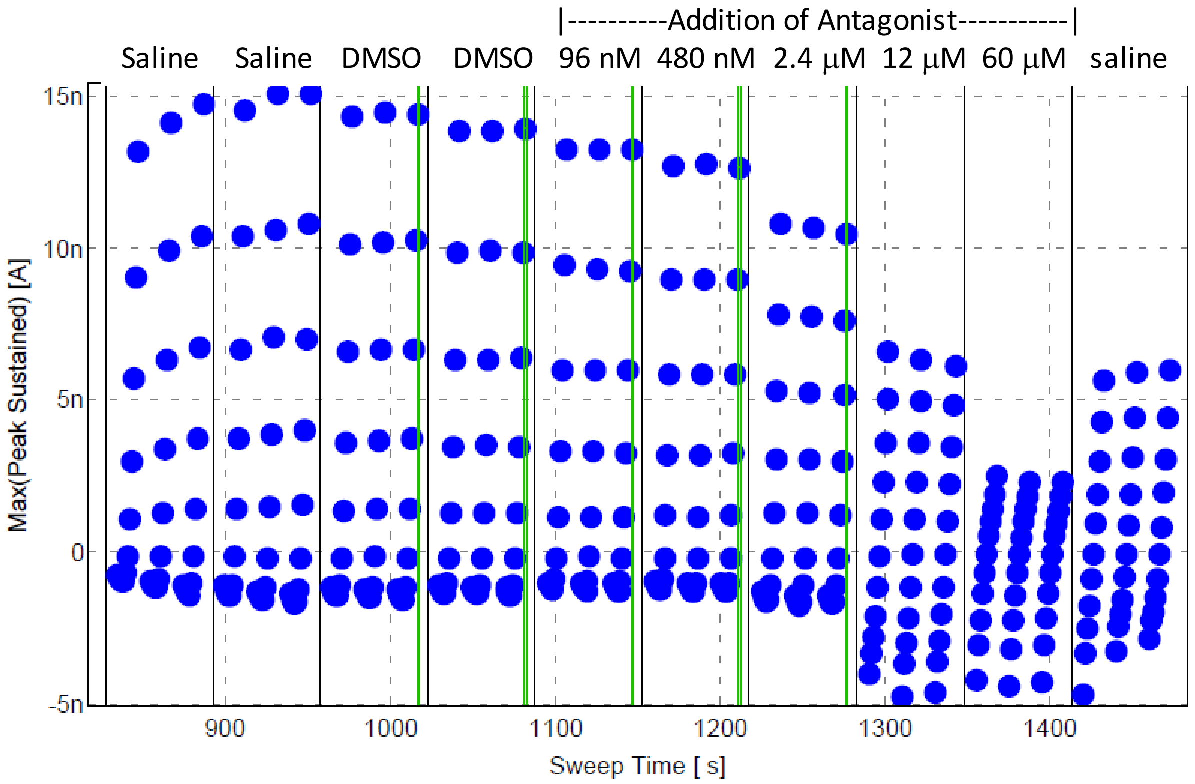

Supplementary Figure 10. **Impact of niflumic acid on TMEM16A (acd) calcium-activated chloride currents as measured by QPatch electrophysiology.** Shown is a representative recording of the impact of progressive (5-fold) increases of niflumic acid on the TMEM16A current-voltage response. Three I/V protocols were run from -100 to +100 mV (20 mV steps) from a Vhold of 0 mV for each sample addition, which allowed at least 60s incubation time per compound dose in our standard QPatch assay for determining potency (see Materials and Methods). The channel exhibited the expected voltage-dependence with these recordings using 170 nM free intracellular calcium. Niflumic acid inhibited the outward current but showed a paradoxical stimulation of inward currents from -60 to -100 mV.

3.11 Supplementary Figure 11

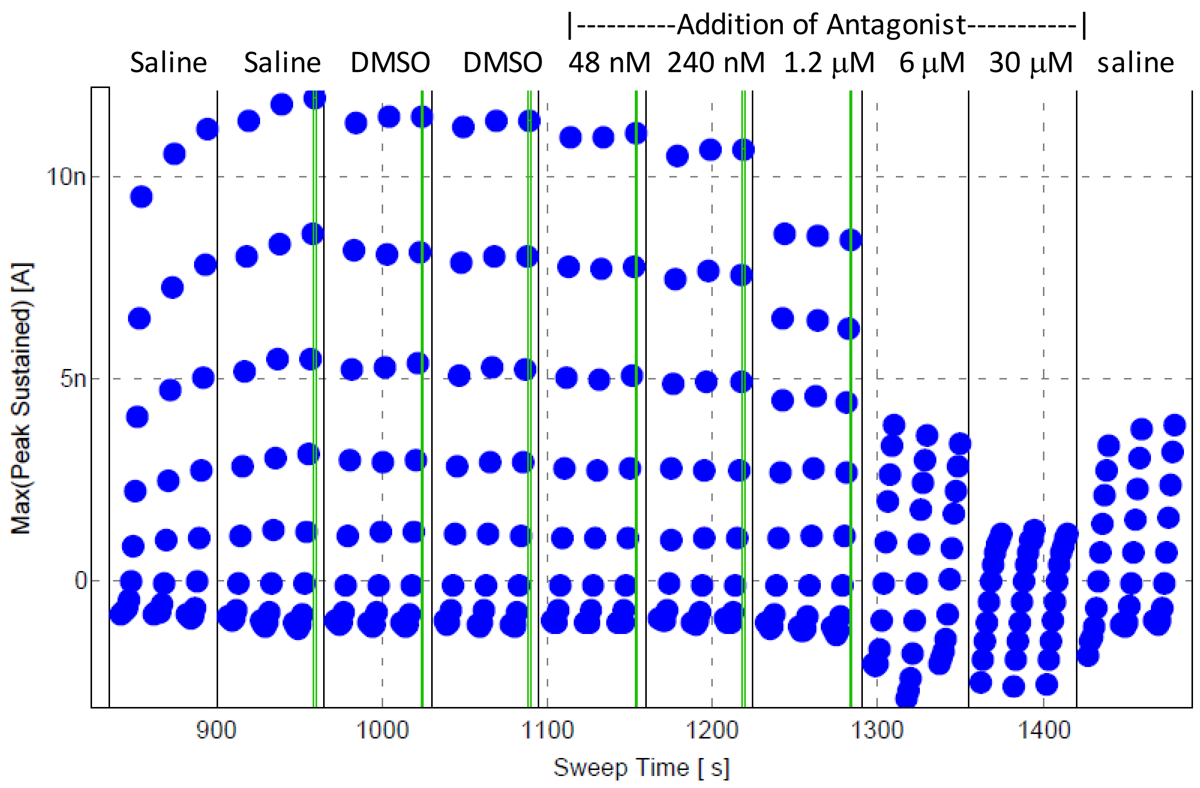

Supplementary Figure 11. **Impact of 1PBC on TMEM16A (acd) calcium-activated chloride currents as measured by QPatch electrophysiology.** Shown is a representative recording of the impact of progressive (5-fold) increases of 1PBC on the TMEM16A current-voltage response. Three I/V protocols were run from -100 to +100 mV (20 mV steps) from a Vhold of 0 mV for each sample addition, which allowed at least 60s incubation time per compound dose in our standard QPatch assay for determining potency (see Materials and Methods). The channel exhibited the expected voltage-dependence with these recordings using 170 nM free intracellular calcium. 1PBC inhibited the outward current but showed a paradoxical stimulation of inward currents from -60 to -100 mV.

3.12 Supplementary Figure 12

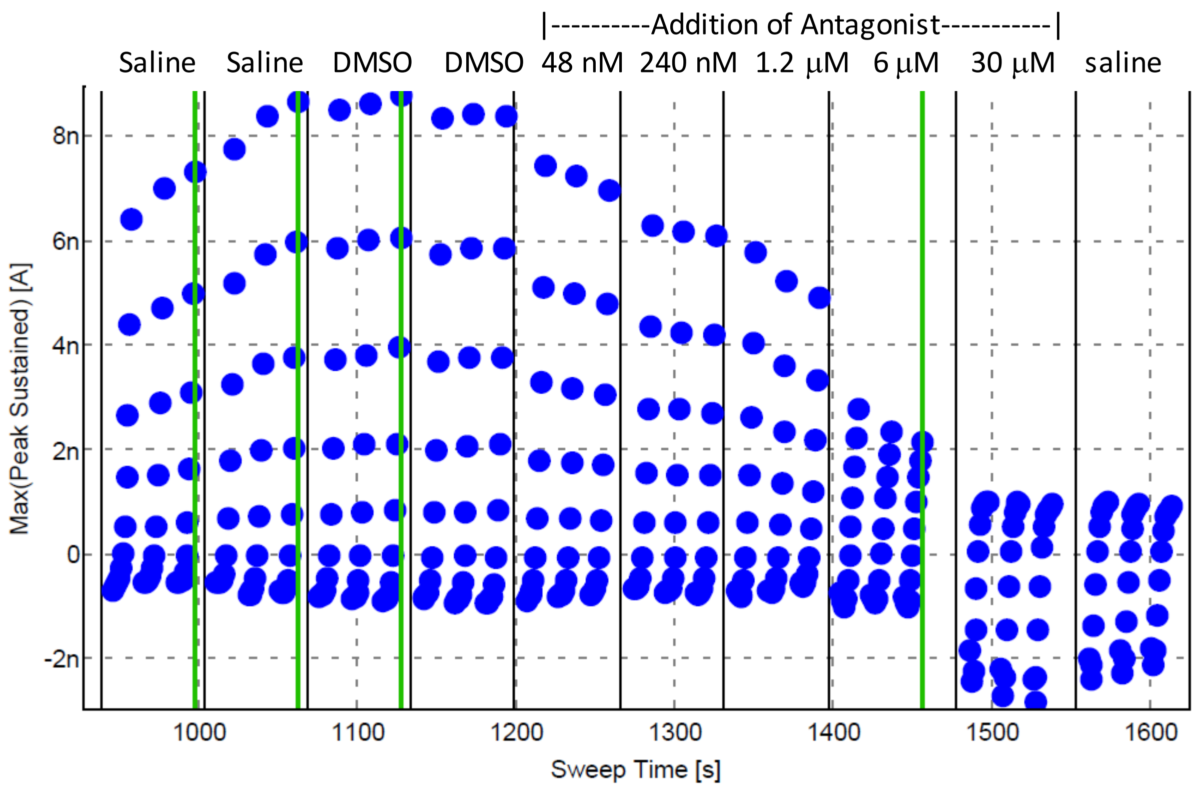

Supplementary Figure 12. **Impact of niclosamide on TMEM16A (acd) calcium-activated chloride currents as measured by QPatch electrophysiology.** Shown is a representative recording of the impact of progressive (5-fold) increases of niclosamide on the TMEM16A current-voltage response. Three I/V protocols were run from -100 to +100 mV (20 mV steps) from a Vhold of 0 mV for each sample addition, which allowed at least 60s incubation time per compound dose in our standard QPatch assay for determining potency (see Materials and Methods). The channel exhibited the expected voltage-dependence with these recordings using 170 nM free intracellular calcium. Niclosamide provided robust inhibition of the outward current but showed a paradoxical stimulation of the inward currents after voltage steps between -60 to -100 mV from a Vhold of 0 mV.

3.13 Supplementary Figure 13

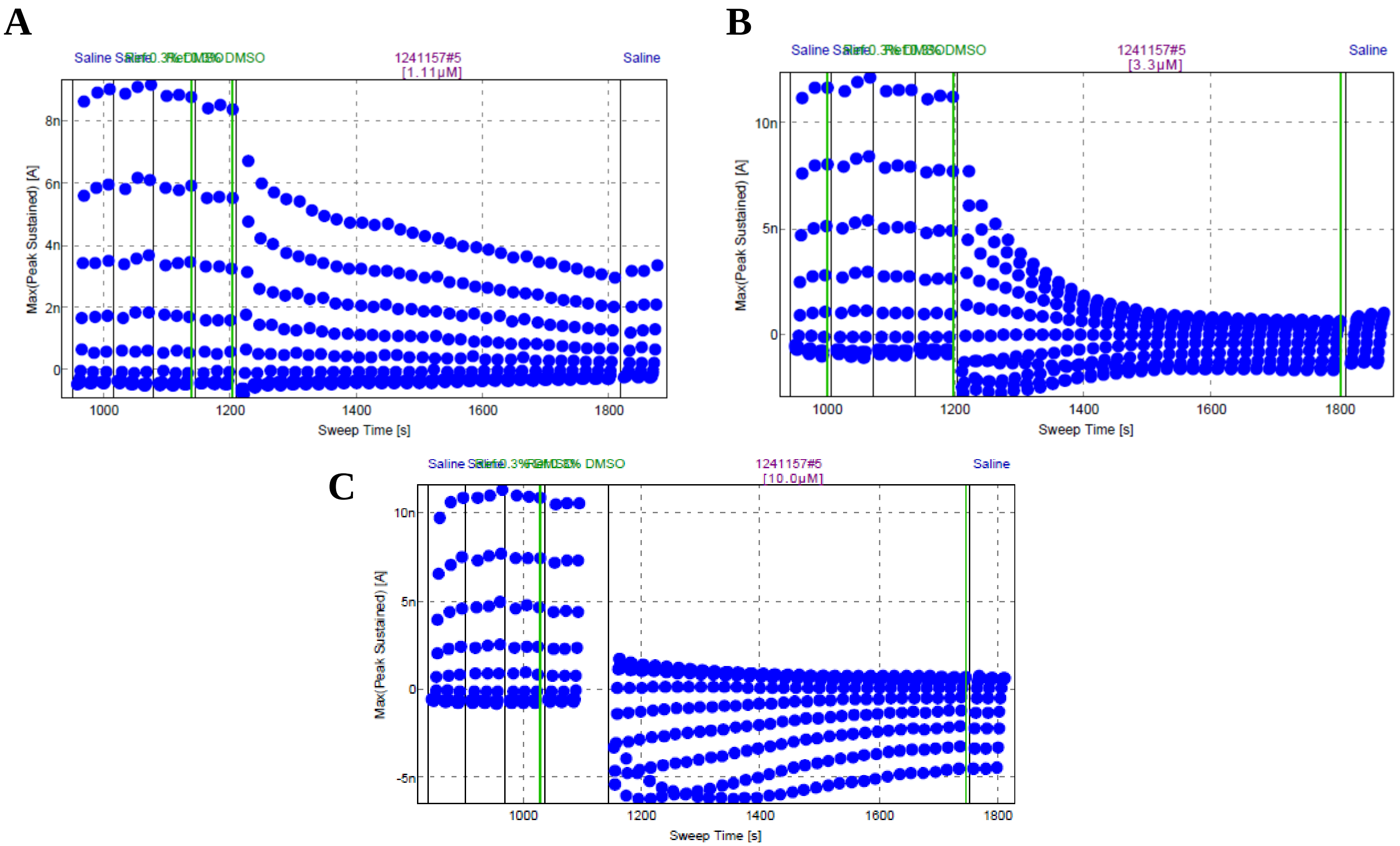

Supplementary Figure 13. **Effect of longer incubation times of niclosamide (Cpd 1, 1241157) on TMEM16A (acd) calcium-activated chloride currents at differing membrane potentials as measured by whole cell patch clamp electrophysiology.** Shown is representative QPatch recordings over time after addition of 1.1 µM **(A)**, 3.3 µM **(B)** and 10.0 µM **(C)** of compound. Using a Vhold of 0 mV, niclosamide decreased the outward currents, but caused a paradoxical increase of the inward currents. The kinetics of niclosamide block of the outward current is delayed at lower concentrations indicating the standard QPatch recording time of ~60s per dose of our medium/high-throughput medchem QPatch assay may underestimate this compound’s potency (see Suppl. Figure 12 and Materials and Methods).

3.14 Supplementary Figure 14

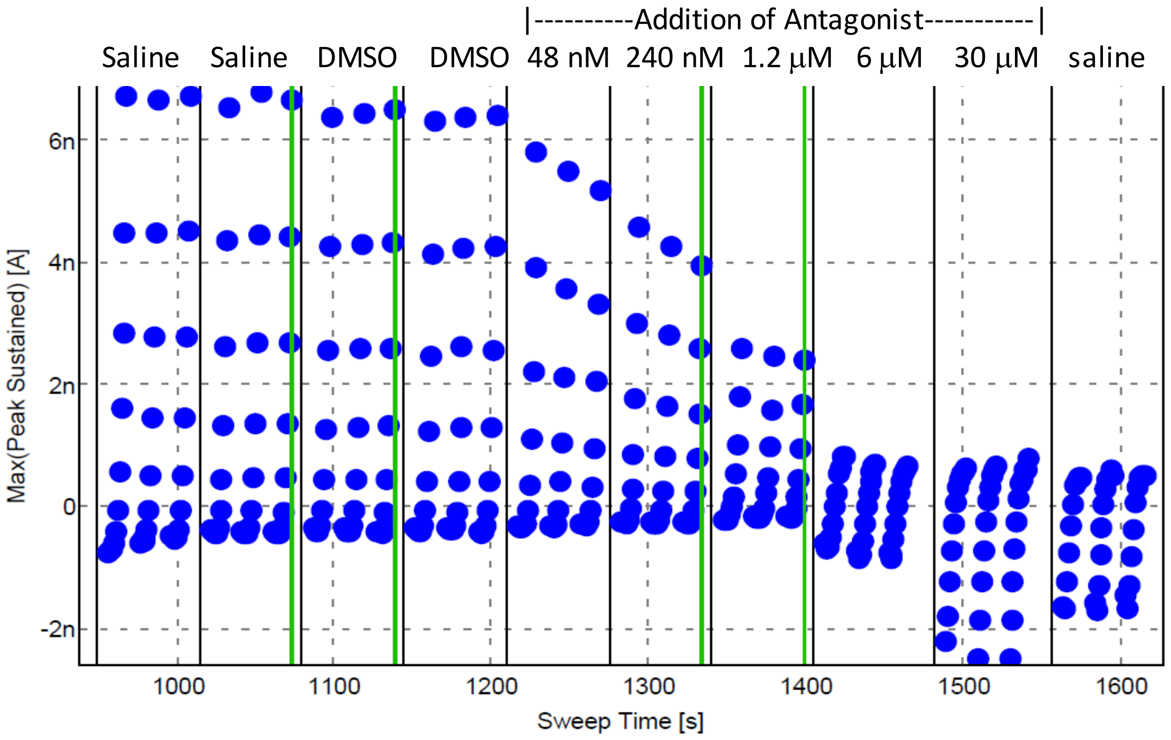

Supplementary Figure 14. **Impact of Compound 2 on TMEM16A (acd) calcium-activated chloride currents as measured by QPatch electrophysiology.** Shown is a representative recording of the impact of progressive (5-fold) increases of Compound 2 on the TMEM16A current-voltage response. Three I/V protocols were run from -100 to +100 mV (20 mV steps) from a Vhold of 0 mV for each sample addition, which allowed at least 60s incubation time per compound dose in our standard QPatch assay for determining potency (see Materials and Methods). The channel exhibited the expected voltage-dependence with these recordings using 170 nM free intracellular calcium. Compound 2 inhibited the outward current but showed a paradoxical stimulation of inward currents.

3.15 Supplementary Figure 15

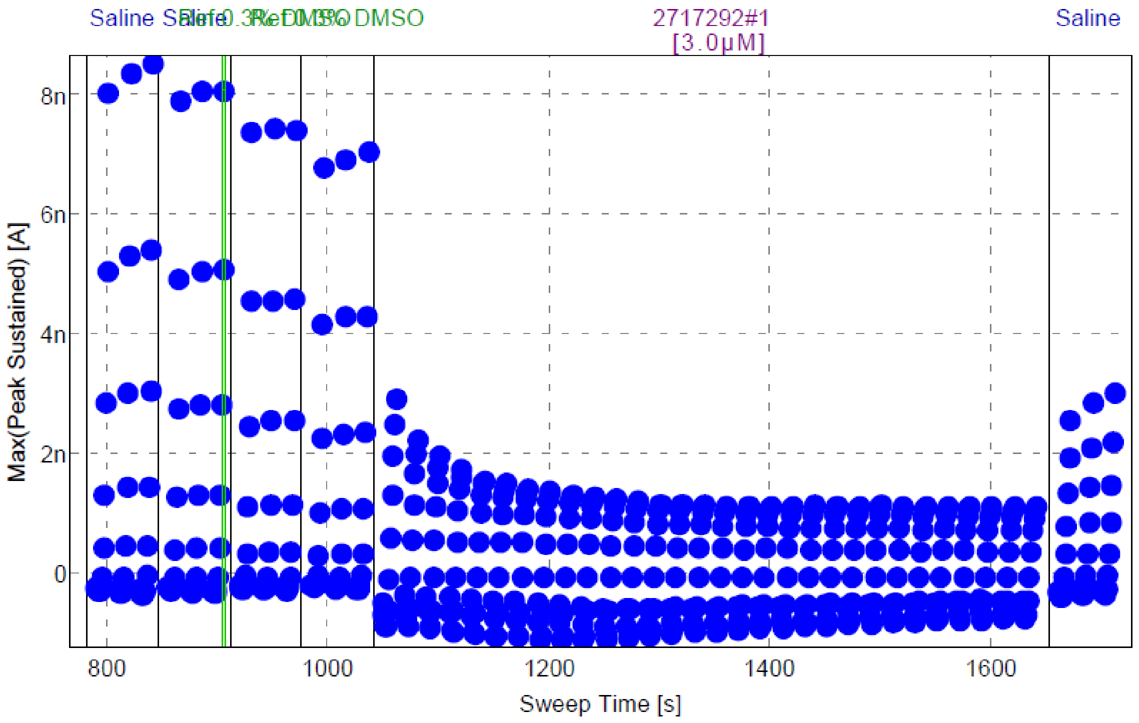

Supplementary Figure 15. **Effect of longer incubation times of Compound 2 (Cpd 2, 2717292) on TMEM16A (acd) calcium-activated chloride currents at different membrane potentials as measured by whole cell patch clamp electrophysiology.** Shown is representative QPatch recordings over time after addition of 3.0 µM Compound 2 using a voltage protocol with a Vhold of 0 mV where membrane potential was changed from -100 to +100 mV in 20 mV steps.

3.16 Supplementary Figure 16

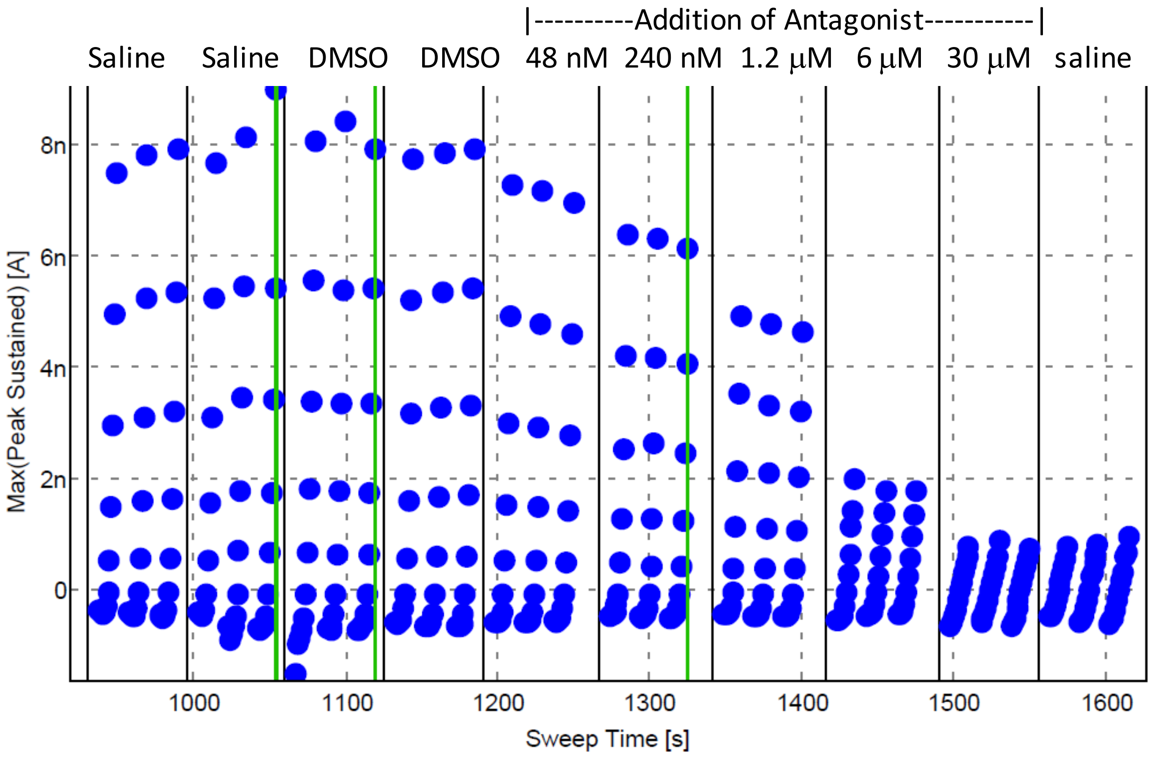

Supplementary Figure 16. **Impact of Compound 4 on TMEM16A (acd) calcium-activated chloride currents as measured by QPatch electrophysiology.** Shown is a representative recording of the impact of progressive (5-fold) increases of Compound 4 on the TMEM16A current-voltage response. Three I/V protocols were run from -100 to +100 mV (20 mV steps) from a Vhold of 0 mV for each sample addition, which allowed at least 60s incubation time per compound dose in our standard QPatch assay for determining potency (see Materials and Methods). The channel exhibited the expected voltage-dependence with these recordings using 170 nM free intracellular calcium. Compound 4 showed robust inhibition of the outward currents, with effects observed even at low concentrations of 48-240 nM.

3.17 Supplementary Figure 17

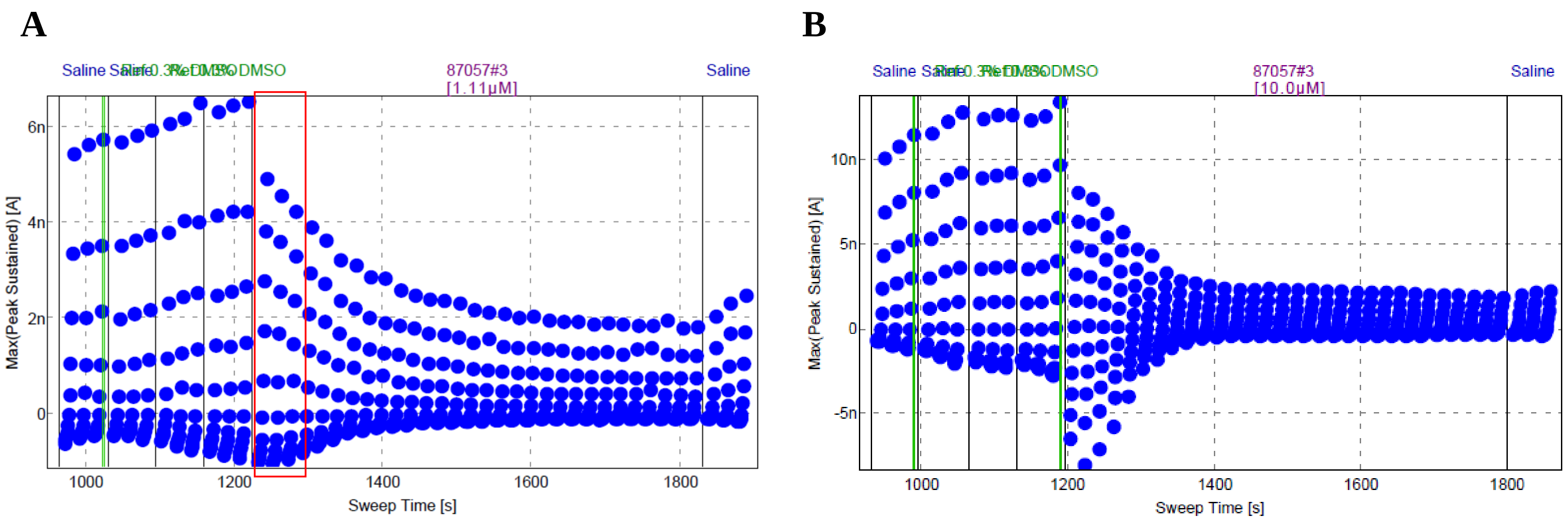

Supplementary Figure 17. **Effect of longer incubation times and repeated pulses on Compound 4 (Cpd 4, 87057) block of TMEM16A (acd) calcium-activated chloride currents as measured by whole cell patch clamp electrophysiology.** Shown are representative QPatch recordings over time after addition of 1.1 µM **(A)** or 10 µM Cpd 4 **(B)**. The red rectangle of panel **(A)** is the typical time frame allowed for each compound dose in our standard cumulative concentration response protocol, which suggests our standard QPatch protocol for medium-/high-throughput medchem support may not allow sufficient time for some compounds to achieve steady-state block of the channel. The kinetics of Cpd 4 at 10 µM reveal a progressive and stable inhibition of the outward current, but transient stimulation of the inward current which resolves over time **(B)**.

3.18 Supplementary Figure 18

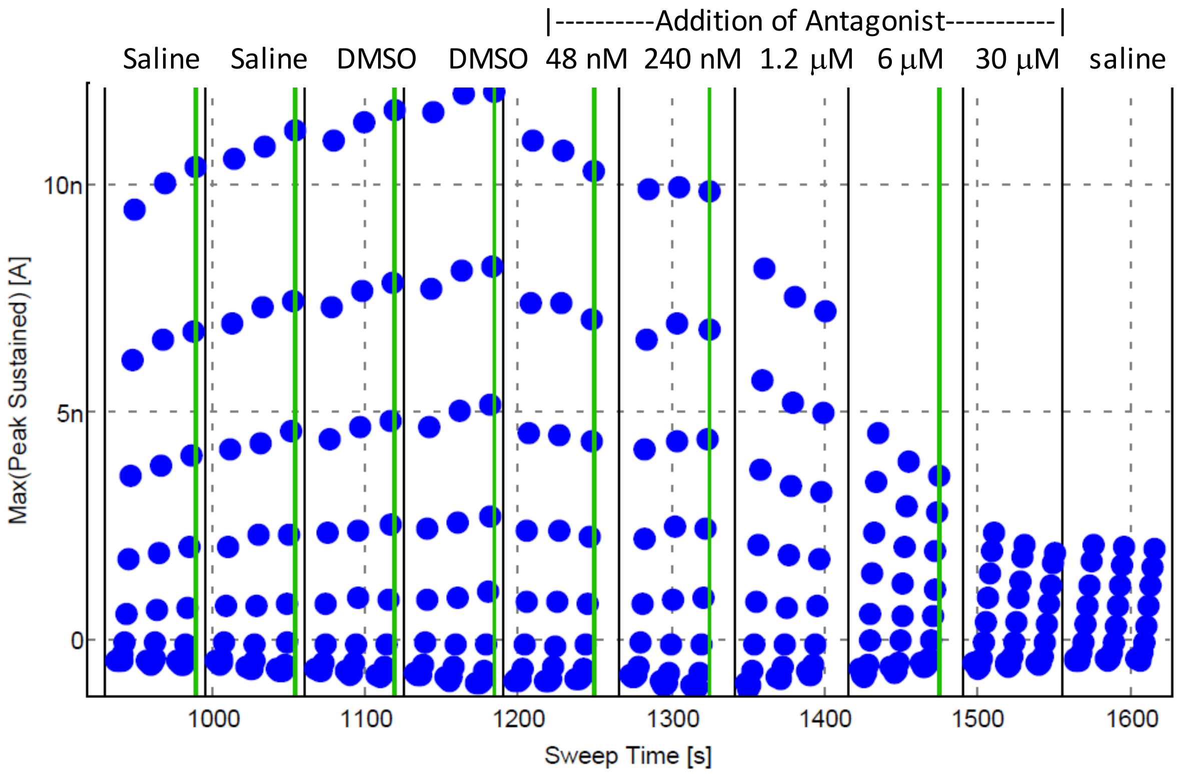

Supplementary Figure 18. **Impact of Compound 3 on TMEM16A (acd) calcium-activated chloride currents as measured by QPatch electrophysiology.** Shown is a representative recording of the impact of progressive (5-fold) increases of Compound 3 on the TMEM16A current-voltage response. Three I/V protocols were run from -100 to +100 mV (20 mV steps) from a Vhold of 0 mV for each sample addition, which allowed at least 60s incubation time per compound dose in our standard QPatch assay for determining potency (see Materials and Methods). The channel exhibited the expected voltage-dependence with these recordings using 170 nM free intracellular calcium. Compound 3 showed robust inhibition of the outward currents, with effects observed even at the lowest concentrations tested.

3.19 Supplementary Figure 19

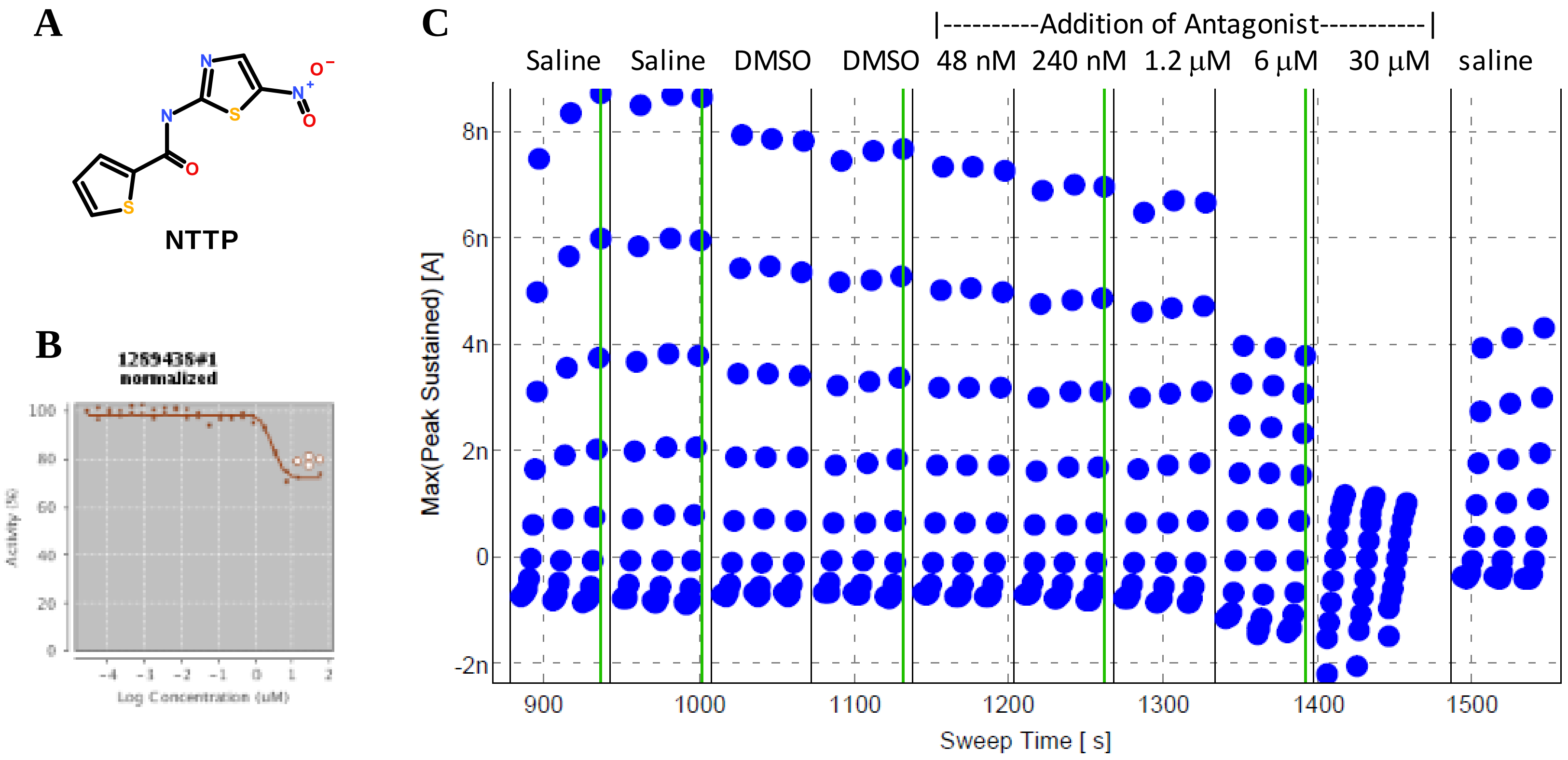

Supplementary Figure 19. **Impact of NTTP (1289438) on TMEM16A iodide eYFP response and chloride currents.** Panel **(A)** provides the structure of NTTP. Dose response results from the TMEM16A (abc) eYFP HTS assay **(B)** indicates NTTP provides potent (IC_{50} = 2.84 μM) but partial block of the ionomycin-induced eYFP quenching using iodide as the permeant anion and NTTP concentrations up to 55.6 μM. In contrast, Panel **(C)** shows in a representative QPatch electrophysiology recording NTTP provides near complete block of the calcium-activated chloride current (IC_{50} = 4.69 μM; n=4). Three I/V protocols were run from -100 to +100 mV (20 mV steps) from a Vhold of 0 mV for each sample addition, which allowed at least 60s incubation time per compound dose in our standard QPatch assay (see Materials and Methods). The channel exhibited the expected voltage-dependence with these recordings using 170 nM free intracellular calcium. NTTP inhibited the outward current but showed a paradoxical stimulation of inward currents from -60 to -100 mV.

3.20 Supplementary Figure 20

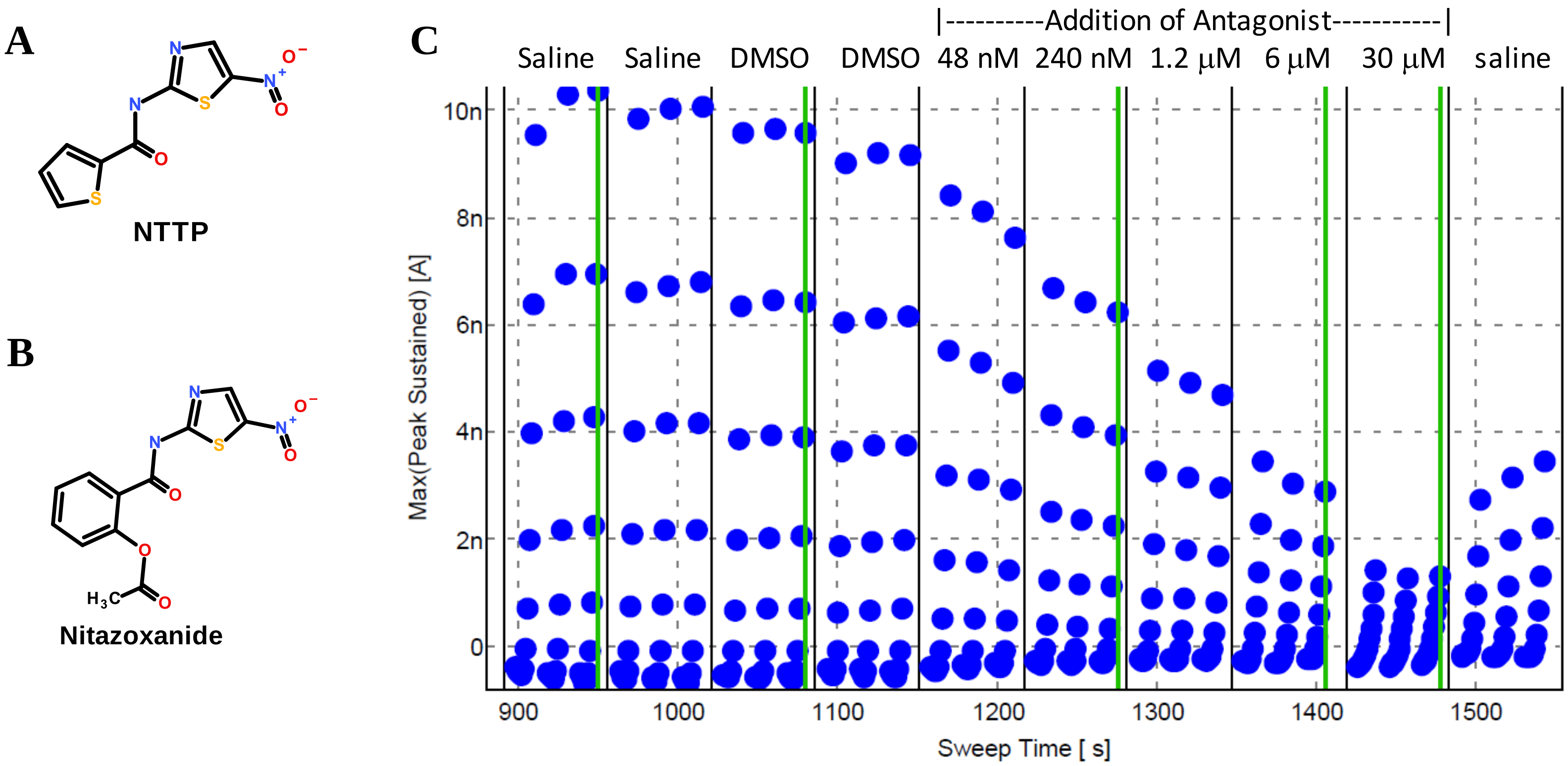

Supplementary Figure 20. **The approved drug nitazoxanide is a TMEM16A antagonist that is structurally similar to NTTP.** The structures of NTTP and nitazoxanide are provided in Panels **(A)** and **(B)**, respectively. Panel **(C)** shows representative results from our QPatch electrophysiology studies evaluating nitazoxanide inhibition of the TMEM16A (acd) calcium-activated chloride current in HEK293 cells after applying progressive (5-fold) increases in drug. The average nitazoxanide IC_{50} from three separate QPatch recordings was 1.26 µM. Shown are three I/V protocols run from -100 to +100 mV (20 mV steps) from a Vhold of 0 mV for each sample addition, which allowed at least 60s incubation time per compound dose in our standard QPatch assay for determining potency (see Materials and Methods). The channel exhibited the expected voltage-dependence with these recordings using 170 nM free intracellular calcium. Nitazoxanide showed robust inhibition of the outward currents, with effects observed even at low concentrations of 48-240 nM.

3.21 Supplementary Figure 21

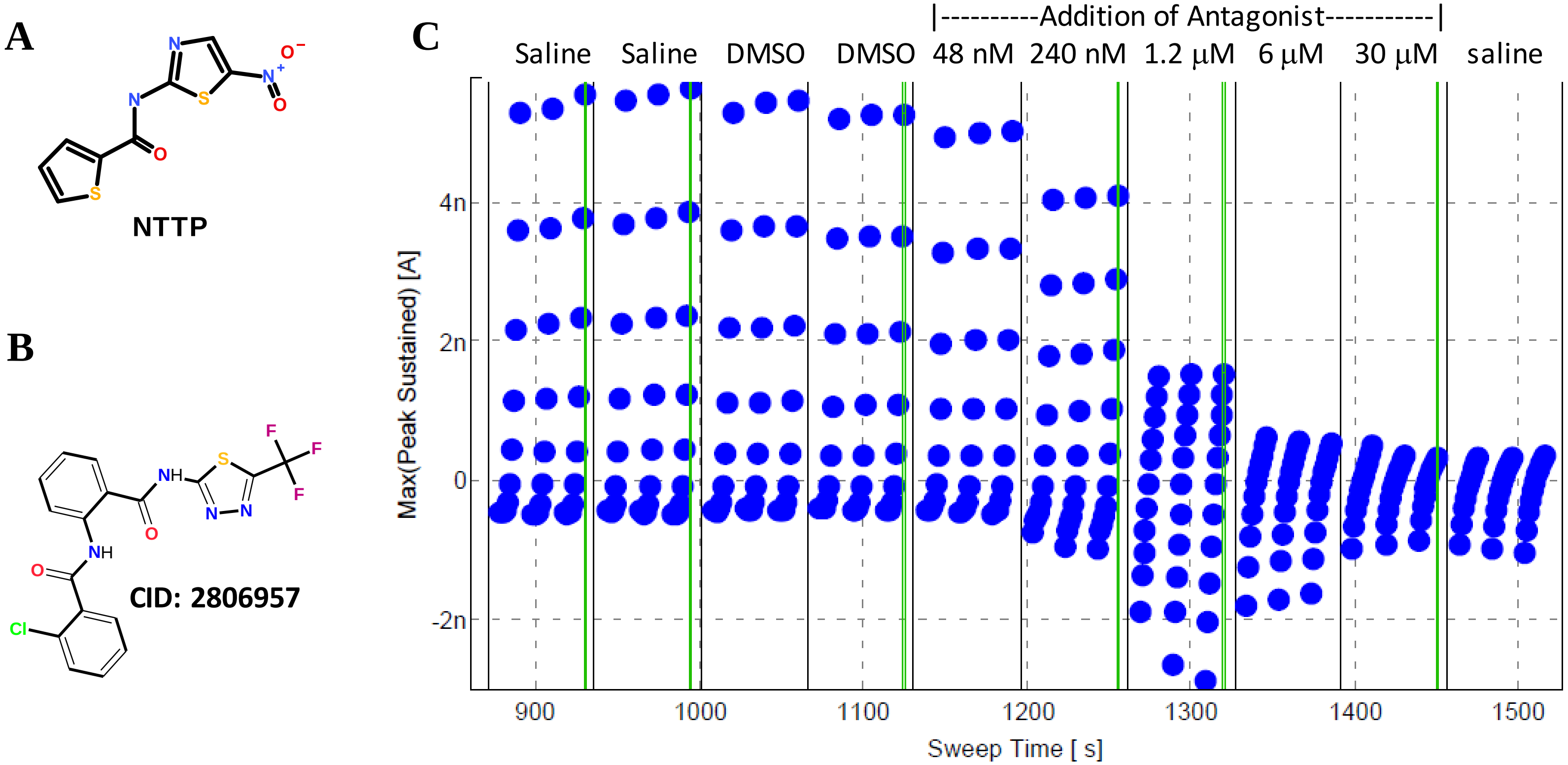

Supplementary Figure 21. **Impact of NTTP-related compound, CID# 2806957 on TMEM16A (acd) calcium-activated chloride currents as measured by QPatch electrophysiology.** Panel **(A)** provides the structure of NTTP, while Panel **(B)** provides the structure of CID# 2806957. A representative recording of progressive (5-fold) increases in concentration of CID# 2806957 on the TMEM16A current-voltage response is shown in Panel **(C)**. This compound provided and average IC$_{50}$ of 0.67 μM (n=3) for blocking the TMEM16A outward current. Three I/V protocols were run from -100 to +100 mV (20 mV steps) from a Vhold of 0 mV for each sample addition, which allowed at least 60s incubation time per compound dose in our standard QPatch assay for determining potency (see Materials and Methods). The channel exhibited the expected voltage-dependence with these recordings using 170 nM free intracellular calcium. CID# 2806957 inhibited the outward current but showed a paradoxical stimulation of inward currents at negative membrane potentials.

3.22 Supplementary Figure 22

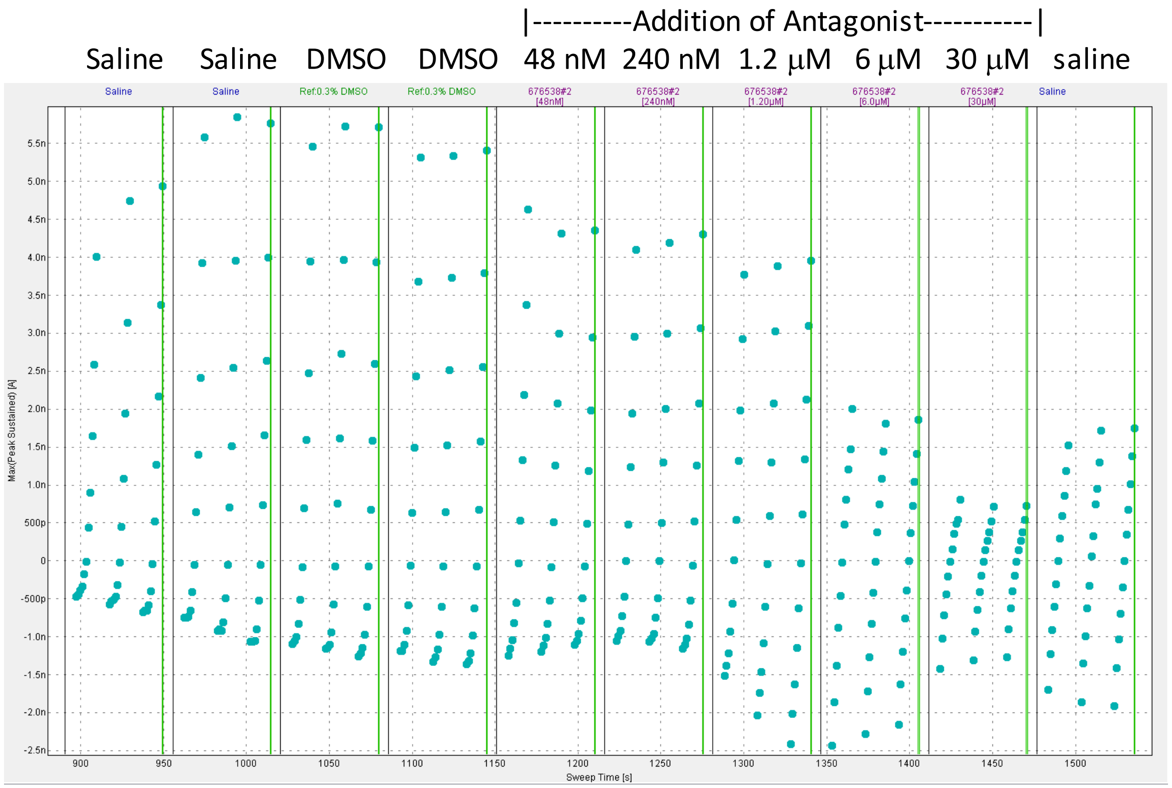

Supplementary Figure 22. **Impact of the TMEM16A antagonist 1PBC (676538) on endogenous calcium-activated chloride currents from colorectal cancer cell line COLO-205 as measured by QPatch electrophysiology.** Shown is raw data from COLO-205 cells measuring the effect of 1PBC on the TMEM16A outward and inward currents. Using cells expressing TMEM16A endogenously, 1PBC caused the same paradoxical inhibition of the outward but stimulation of the inward current as observed earlier using HEK293 cells stably expressing the cloned TMEM16A (acd) variant (see Supplementary Figure 11). Three I/V protocols were run from -100 to +100 mV (20 mV steps) from a Vhold of 0 mV per sample addition which allowed at least 60s incubation time at each compound dose (see Materials and Methods section). Using low free intracellular calcium (170 nM), the currents exhibited the expected voltage-dependence of TMEM16A.

3.23 Supplementary Figure 23

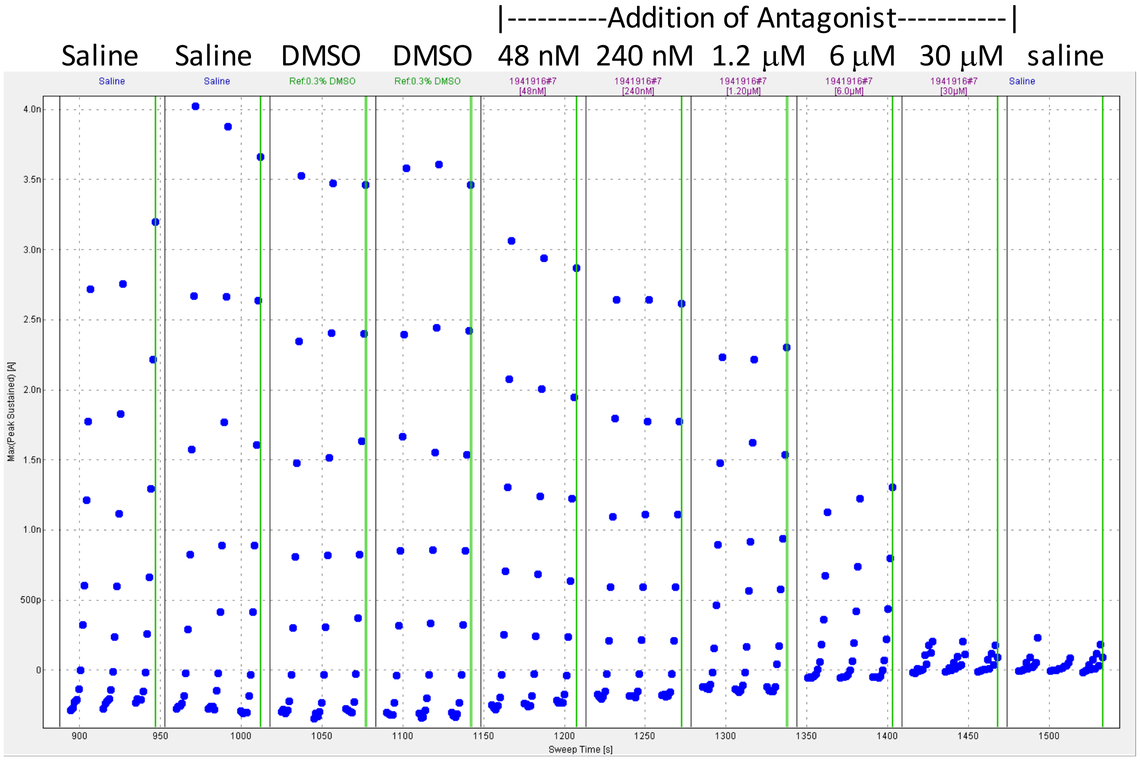

Supplementary Figure 23. **Impact of the TMEM16A antagonist benzbromarone on endogenous calcium-activated chloride currents from colorectal cancer cell line COLO-205 as measured by QPatch electrophysiology.** Shown is raw data from a QPatch recording measuring concentration dependent effects on the TMEM16A current-voltage response. Three I/V protocols were run from -100 to +100 mV (20 mV steps) from a Vhold of 0 mV per sample addition which allowed at least 60s incubation time at each compound dose (see Materials and Methods section). Using low free intracellular calcium (170 nM), the currents exhibited the expected voltage-dependence of TMEM16A. Benzbromarone inhibited both the outward and inward currents.

3.24 Supplementary Figure 24

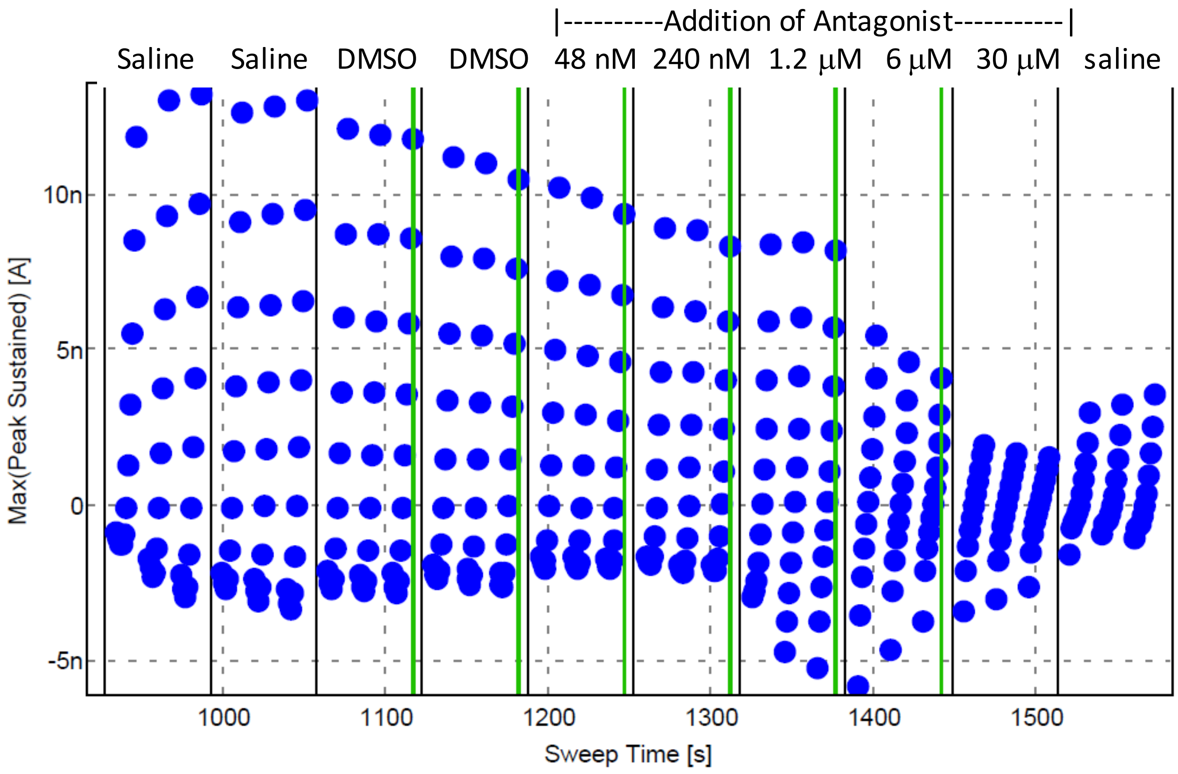

Supplementary Figure 24. **QPatch electrophysiology study of impact 1PBC on TMEM16A (abc) splice variant expressed in HEK293 cells.** Shown is raw data from a recording measuring the effect of 1PBC on the TMEM16A (abc) outward and inward currents. Using cells expressing the (abc) splice variant, 1PBC caused the same paradoxical inhibition of the outward but stimulation of inward current as observed earlier with the (acd) variant (see Supplementary Figure 11). Three I/V protocols were run from -100 to +100 mV (20 mV steps) from a Vhold of 0 mV per sample addition which allowed at least 60s incubation time at each compound dose and recordings used a low free intracellular calcium concentration of 170 nM (see Materials and Methods section).

3.25 Supplementary Figure 25

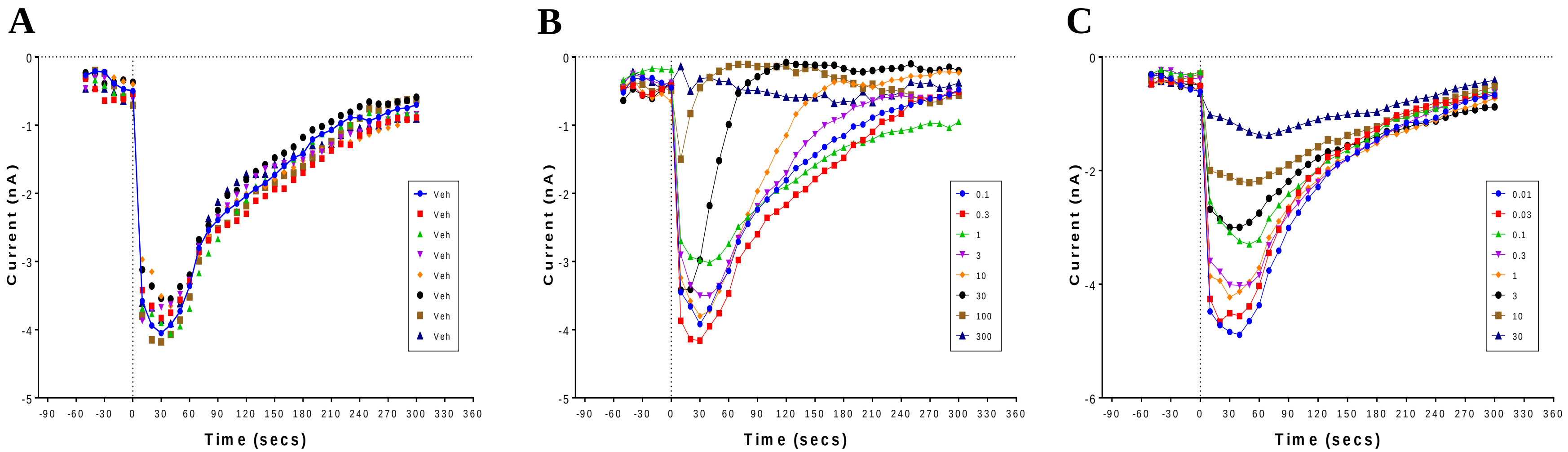

Supplementary Figure 25. **Niflumic acid and benzbromarone inhibit the ionomycin-induced inward current at -100 mV from HEK293 cells stably expressing the TMEM16A (abc) splice variant.** The inward current over time is shown following the co-addition (time 0) of ionomycin (10 µM) with the DMSO vehicle control **(A)**, or various concentration of the antagonists benzbromarone **(B)** or niflumic acid **(C)** in perforated patch clamp electrophysiology studies using the IonWorks Barracuda. Benzbromarone (0.1 – 300 µM) and niflumic acid (0.01 – 30 µM) caused a concentration dependent inhibition of the inward current. The niflumic acid inhibition of the inward currents at -100 mV shown here by perforated patch clamp electrophysiology, contrasts markedly with the paradoxical stimulation shown earlier by QPatch planar whole cell patch clamp studies (see Supplementary Figure 10). Additionally, these perforated patch clamp studies reveal cloned TMEM16A currents in HEK293 cells show the same rapid inactivation described earlier for endogenous calcium-activated chloride currents recorded from tracheal smooth muscle cells following ionomycin or caffeine addition using similar perforated patch clamp electrophysiology methods; see (Wang and Kotlikoff 1997).

3.26 Supplementary Figure 26

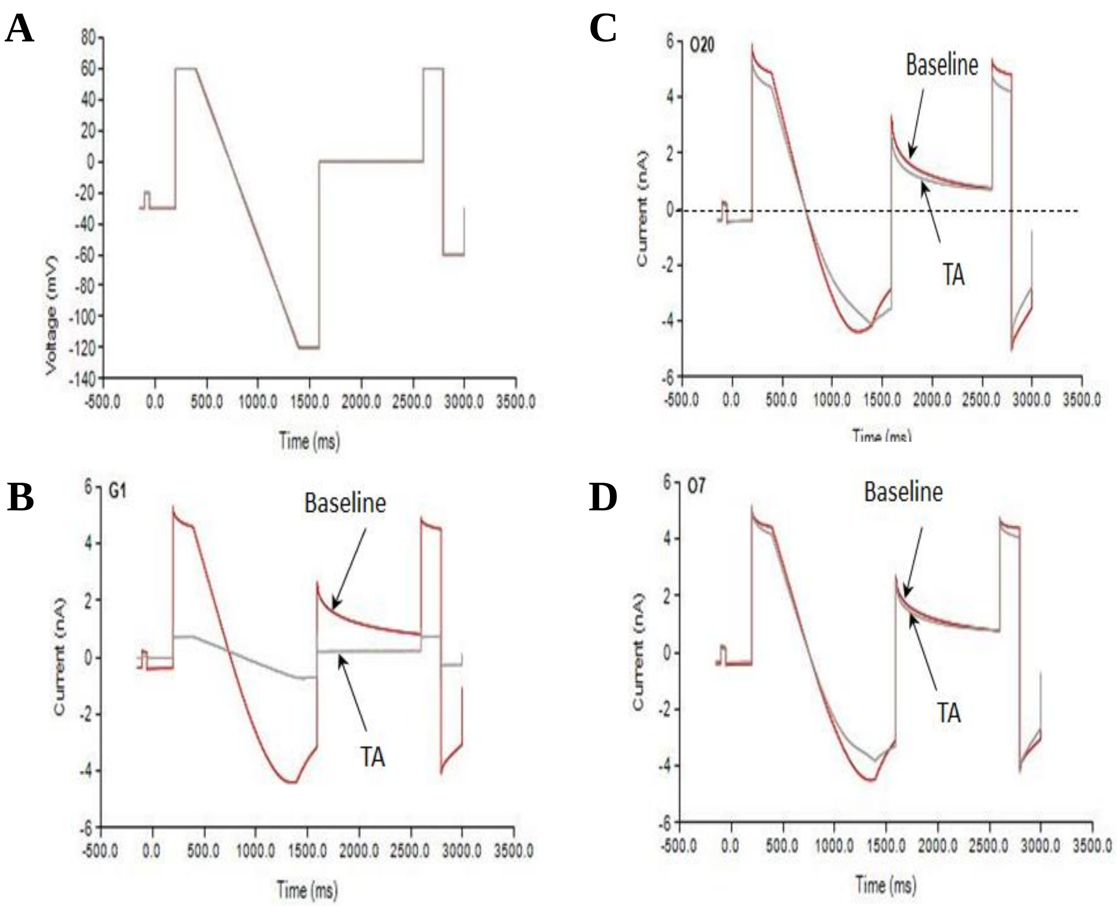

Supplementary Figure 26. **Niclosamide and Compound 4 do not impact the CFTR chloride current.** Panel **(A)** provides the voltage protocol for IonWorks Barracuda electrophysiology studies using CHO cells stably expressing the human CFTR gene. Red traces **(B-D)** show the baseline current, while grey traces show the current following addition of the Test Article (TA) or compound. While 10 µM of the benchmark inhibitor, CFTRinh-172, fully inhibited the cAMP-activated chloride current **(B)**, there were no significant effects on the CFTR chloride current following addition of 10 µM niclosamide **(C)** or 10 µM Compound 4 **(D)**.

3.27 Supplementary Figure 27

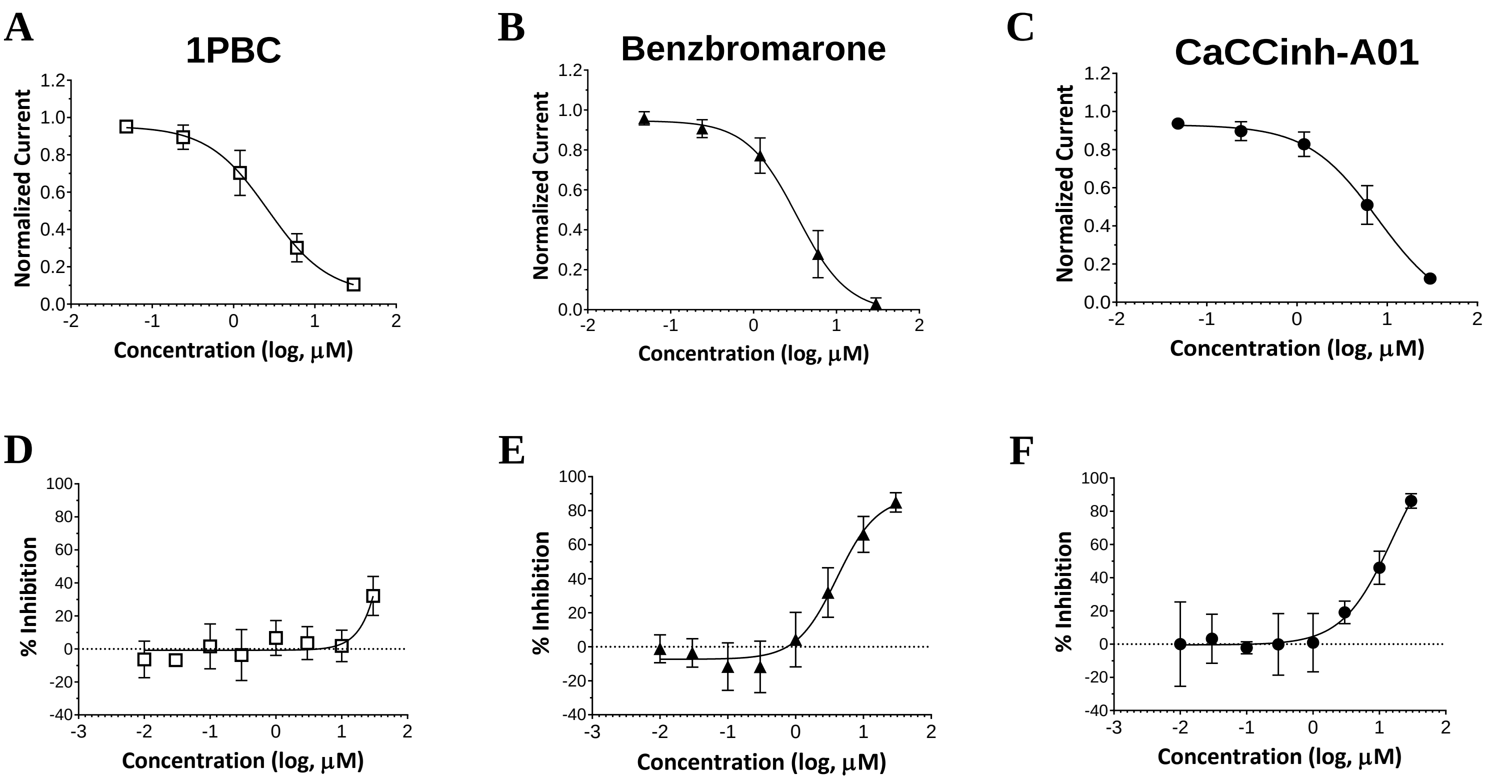

Supplementary Figure 27. **Selectivity of benchmark TMEM16A antagonists in inhibiting TMEM16A (top panels) versus CFTR (bottom panels) chloride currents.** The dose-response results in panels **A-C** are from QPatch electrophysiology studies using HEK293 / TMEM16A (acd) cells and 170 nM free intracellular calcium, while panels **D-F** show the results from IonWorks Barracuda studies using CHO / CFTR cells activated with 20 μM forskolin. Data shown are the mean and standard deviation of 6-7 recordings **(A-C)** or 3-4 recordings **(D-F)**. Of the three TMEM16A antagonists shown, 1PBC appears the most selective in inhibiting TMEM16A vs CFTR.

3.28 Supplementary Figure 28

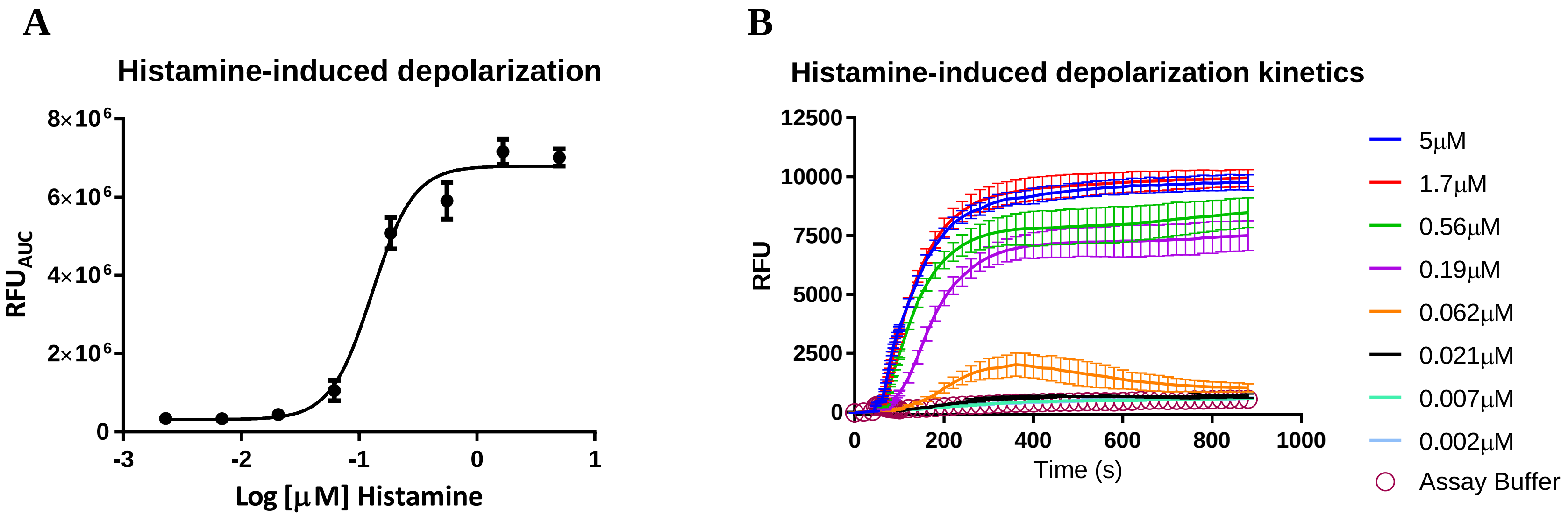

Supplementary Figure 28. **Representative histamine dose-response study in the FLIPR membrane potential assay using cultured primary human airway smooth muscle cells to determine the sensitivity to contractile agents. (A)** Histamine induced a dose-dependent increase in fluorescence corresponding to ASM depolarization (Mean ± SD, N = 4), a response which was sustained as shown in the representative kinetic traces provided in panel **(B)** of the fluorescent response following treatment with different concentrations of histamine. Routine experiments with TMEM16A antagonists involved pre-incubation of cells with compound for ~30 minutes and then treatment with an EC_{90} concentration of histamine. RFU, relative fluorescence units. AUC, area under curve.

3.29 Supplementary Figure 29

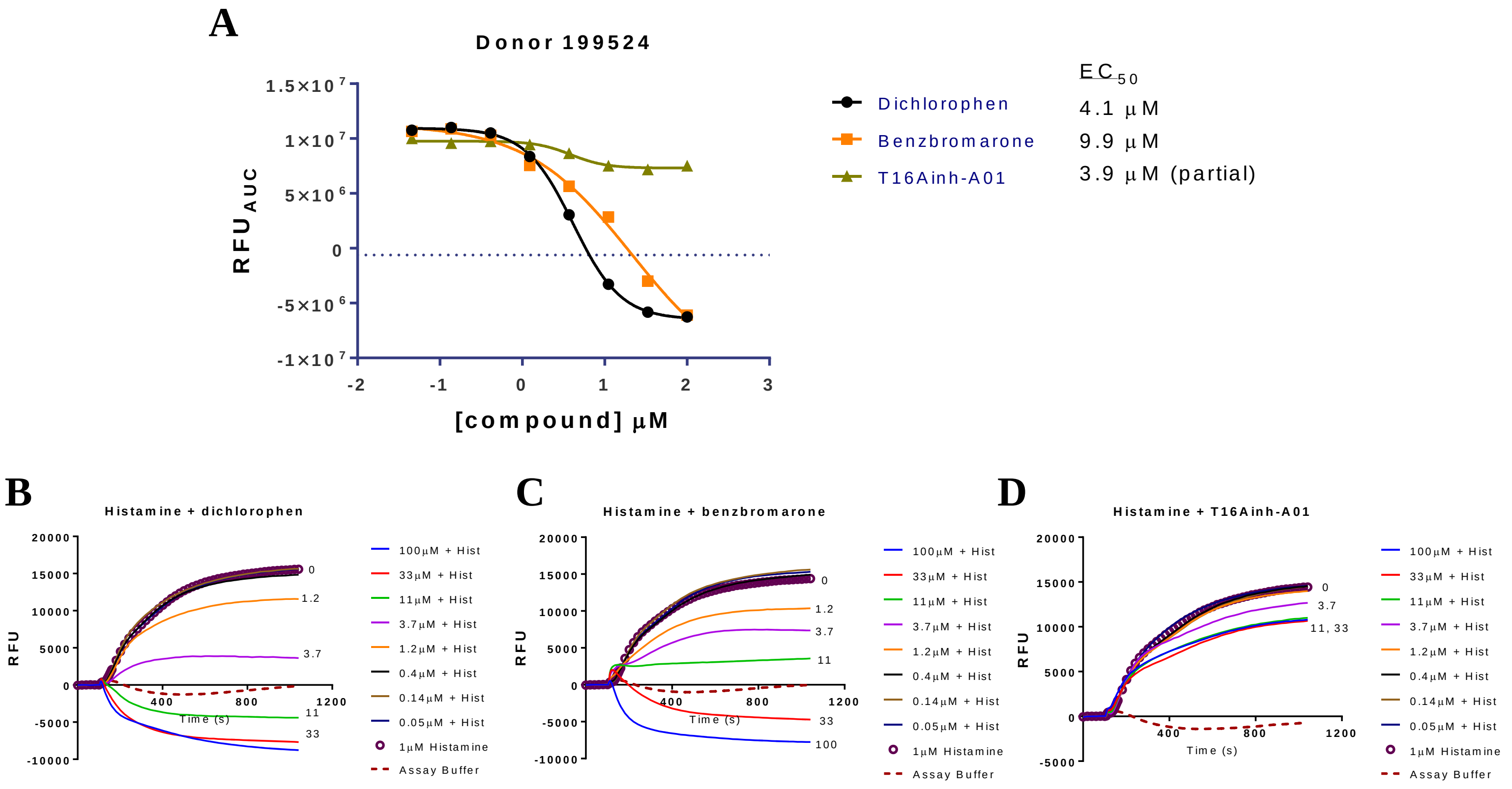

Supplementary Figure 29. **The TMEM16A antagonists dichlorophen and benzbromarone fully inhibit the *histamine-induced* depolarization of primary human airway smooth muscle cells (donor 199524), while T16Ainh-A01 shows just partial effects.** Panels **(B-D)** show raw fluorescence data from the membrane potential assay of buffer alone (dashed line), buffer plus 1 µM histamine (open circles) and the combination of various concentrations of dichlorophen **(B)**, benzbromarone **(C)** and T16Ainh-A01 **(D)** ranging from 0.05 – 100 µM with histamine (solid lines, labeled). Panel **(A)** plots the dose-dependent changes in area under the curve (AUC) for the TMEM16A antagonists. Dichlorophen and benzbromarone fully reversed histamine depolarization and induced some hyperpolarization. RFU = relative fluorescence units.

3.30 Supplementary Figure 30

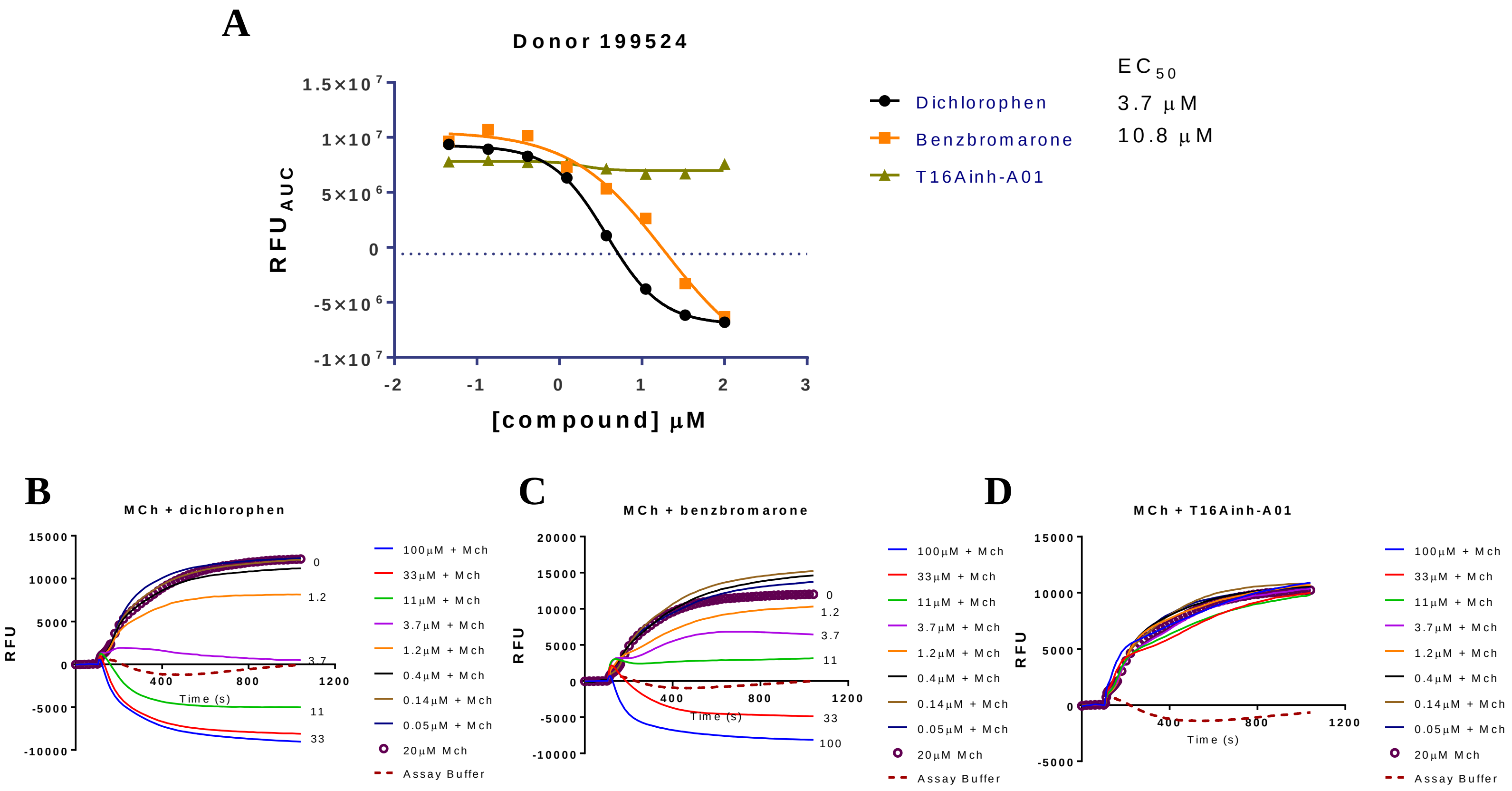

Supplementary Figure 30. **The TMEM16A antagonists dichlorophen and benzbromarone fully inhibit the *methacholine (Mch)-induced* depolarization of primary human airway smooth muscle cells (donor 199524), while T16Ainh-A01 showed little effect.** Panels **(B-D)** show raw fluorescence data from the membrane potential assay of buffer alone (dashed line), buffer plus 20 µM methacholine (open circles) and the combination of various concentrations of dichlorophen **(B)**, benzbromarone **(C)** and T16Ainh-A01 **(D)** ranging from 0.05 – 100 µM with methacholine (solid lines, labeled). Panel **(A)** plots the dose-dependent changes in area under the curve (AUC) of TMEM16A antagonists. Dichlorophen and benzbromarone fully reversed methacholine depolarization and induced some hyperpolarization. RFU = relative fluorescence units.

3.31 Supplementary Figure 31

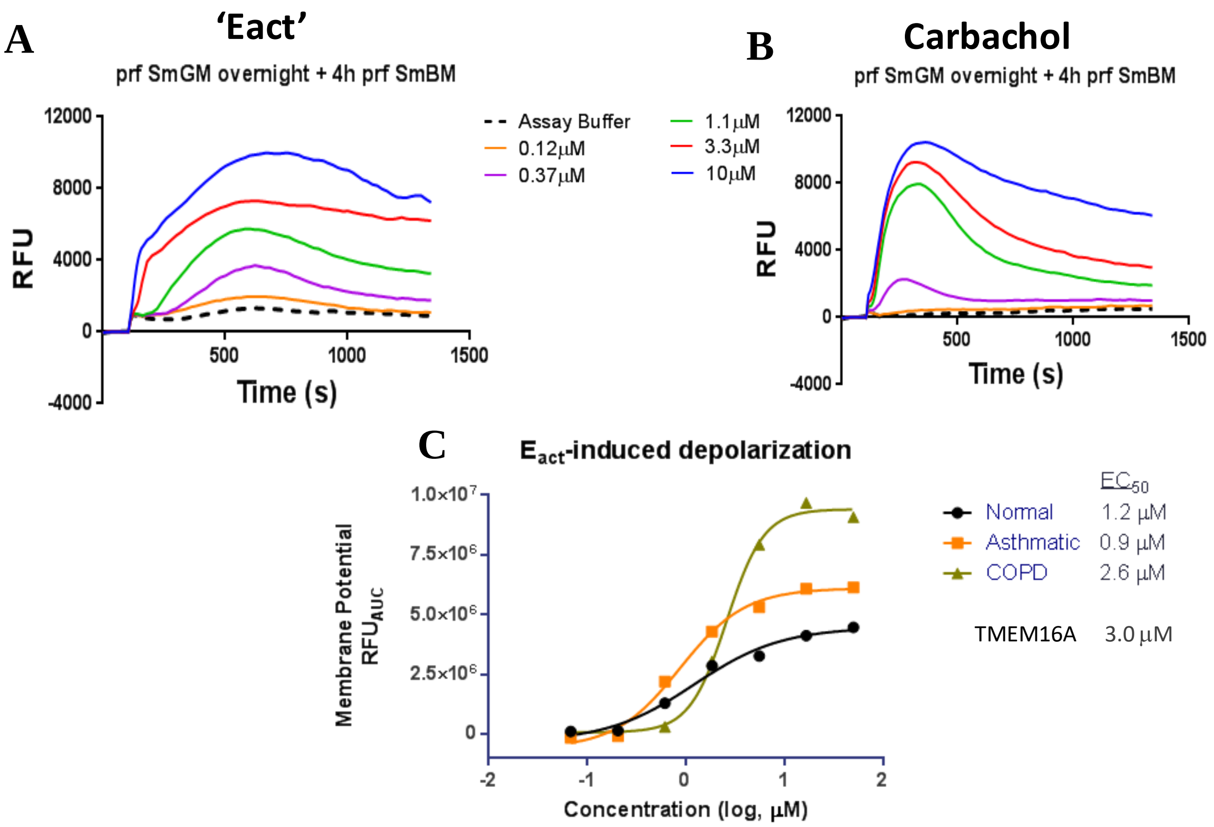

Supplementary Figure 31. The small molecule TMEM16A activator, E_{act}, induces depolarization of primary human airway smooth muscle cells like the contractant carbachol. Kinetic traces from the FLIPR membrane potential assay indicate E_{act} **(A)** and carbachol **(B)** elicit a concentration dependent depolarization of human ASM cells which were serum starved for 4 hours [prf SmGM overnight + 4h prf SmBM]. The potency for E_{act} depolarization of ASM cells isolated from a normal, asthmatic or COPD patient is shown in panel **(C)**. The average EC_{50} of 1 – 3 μM for depolarizing human ASM cells **(C)** compares favorably to the 3.0 μM EC_{50} which Namkung et al. (2011) report for activation of TMEM16A. RFU, relative fluorescence units. AUC, area under the curve.

3.32 Supplementary Figure 32

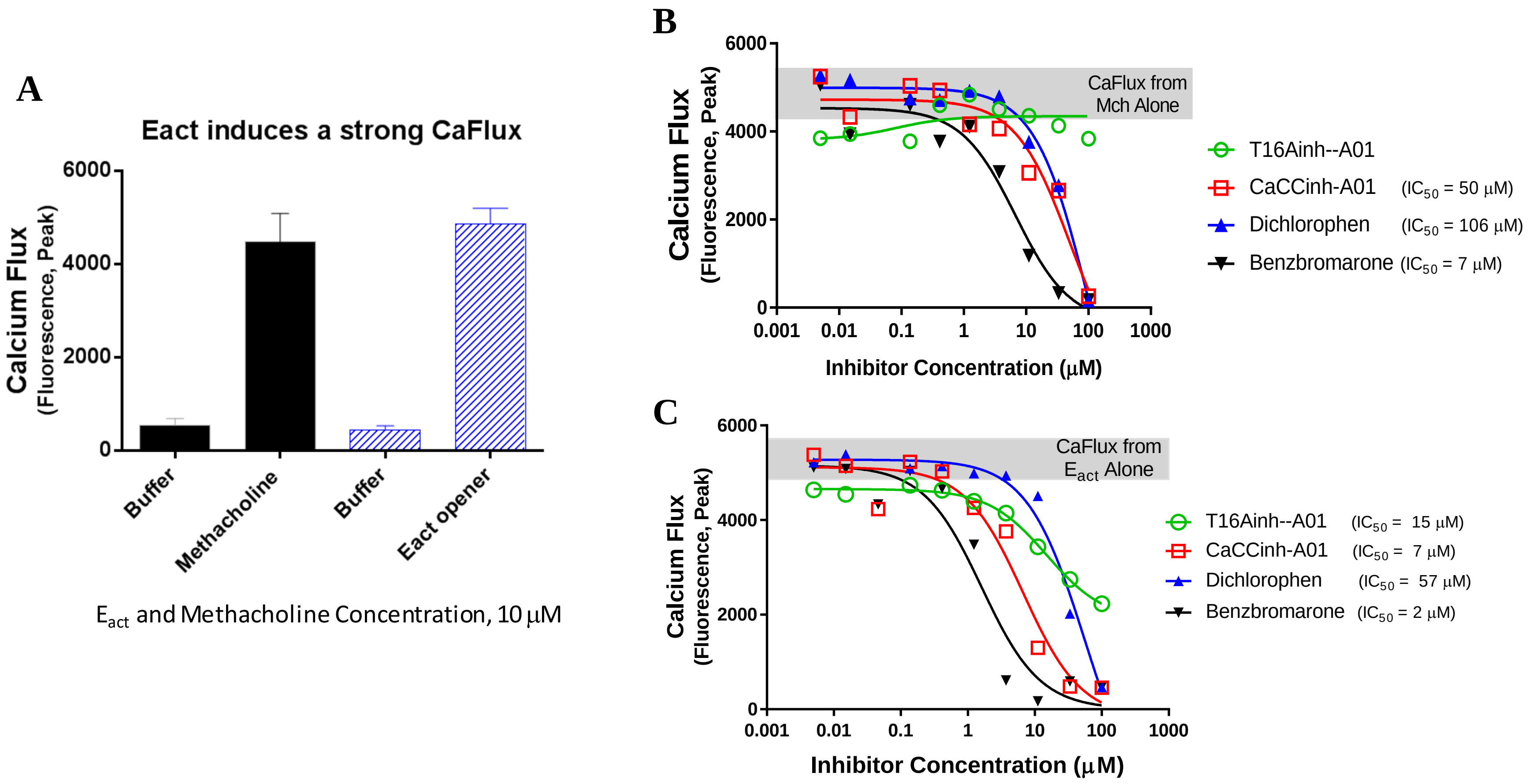

Supplementary Figure 32. **Primary human ASM cells treated with the TMEM16A opener, E_{act}, and the cholinergic methacholine (Mch) cause similar increases in the level of pro-contractile intracellular calcium (A)**. The TMEM16A antagonists benzbromarone, CaCCinh-A01 and dichlorophen block both the Mch **(B)** and E_{act} **(C)** ASM calcium flux (CaFlux), while the TMEM16A antagonist T16Ainh-A01 only showed effects in blocking the CaFlux induced by E_{act}.

3.33 Supplementary Figure 33

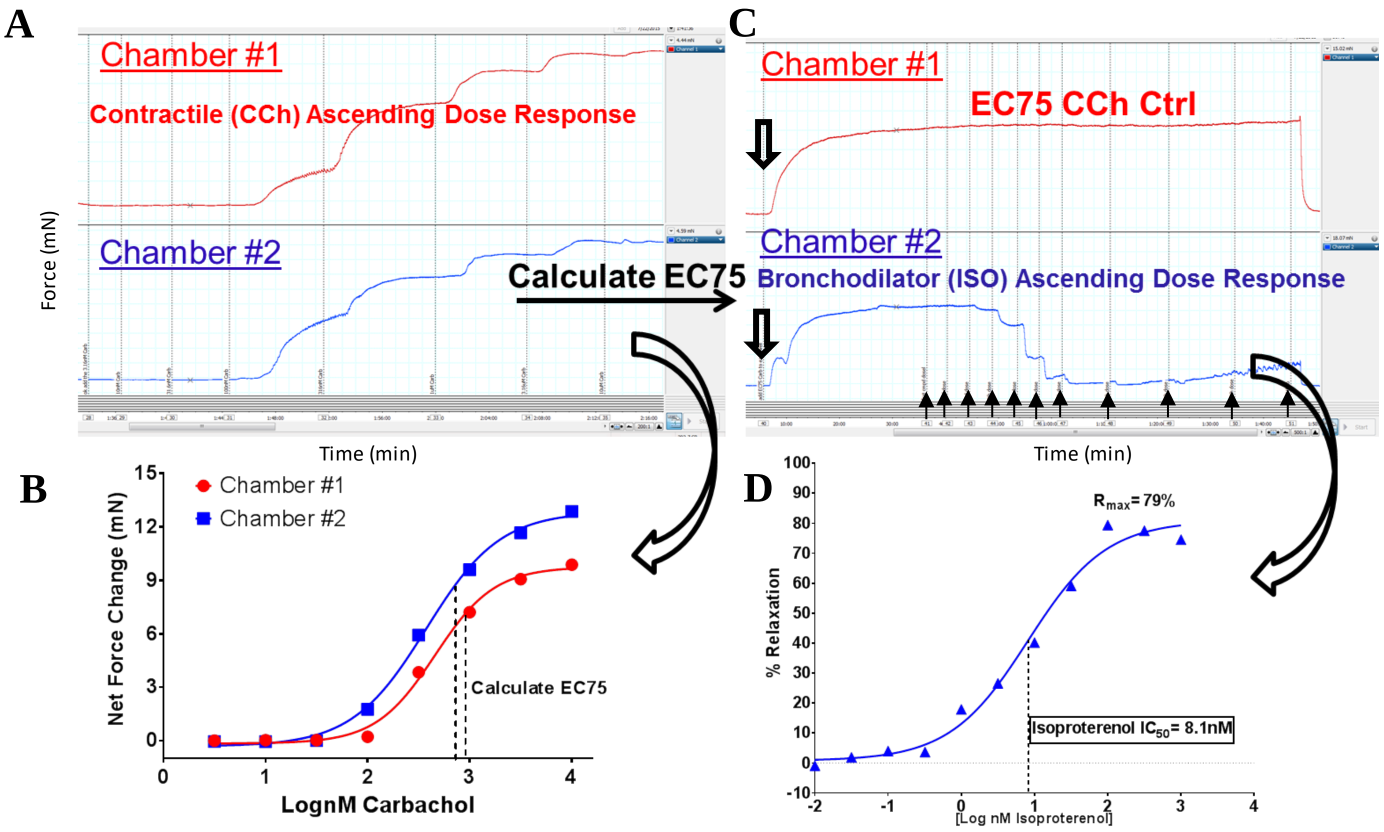

Supplementary Figure 33. **Experimental procedure for determining the EC_{75} of carbachol for contraction of mouse tracheal rings and an example of the efficacy of isoproterenol in relaxing carbachol pre-contracted airways.** Panels **(A)** shows raw traces from the wire myograph studies on two tracheal rings showing ascending carbachol (CCh) concentrations (vertical dotted lines) cause progressive increases in force over time (each light blue block corresponds to 1 min). The calculated EC_{75} for carbachol are shown in panel **(B)**. After washing tissue and resetting baseline tension, the upper trace of panel **(C)** shows raw traces upon addition of an EC_{75} level of CCh alone (open down arrows), while the lower trace of panel **(C)** shows force from EC_{75} of carbachol followed by level of relaxation following increasing doses (up arrows) of isoproterenol. Carbachol causes sustained contraction over time of the tracheal rings **(C)** (each light blue block corresponds to 2 min) and isoproterenol induced potent relaxation which is plotted in panel **(D)**.

3.34 Supplementary Figure 34

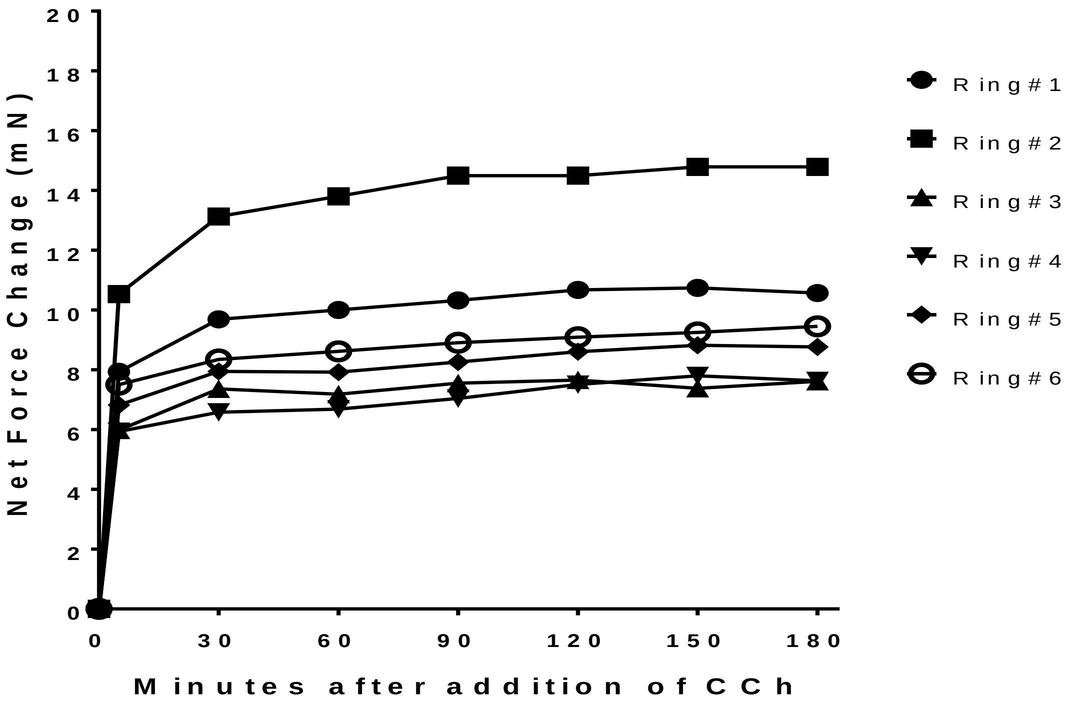

Supplementary Figure 34. **Mouse tracheal rings do not fatigue and maintain contraction for several hours.** DMT wire myograph study on six tracheal rings showing force changes over time following addition of an EC_{75} concentration of carbachol. The contraction remained stable for the entire three-hour recording period shown.

3.35 Supplementary Figure 35

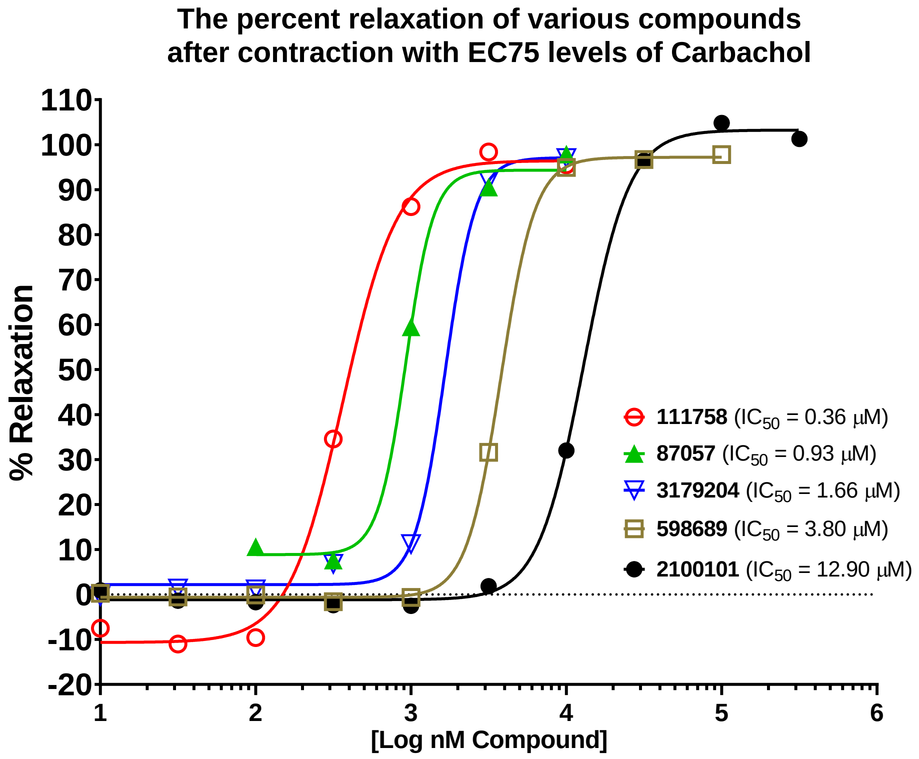

Supplementary Figure 35. **Efficacy of novel TMEM16A antagonists in bronchodilating mouse tracheal rings pre-contracted with carbachol.** Compound 111758 is a structurally related analog of Compound 4 (87057), while 3179204 and 598689 are of the niclosamide series, with the former being a closely related analog of Compound 5. The compound 2100101 is a distinct chemotype from niclosamide or the Compound 4 series. The EC_{50} of compounds in bronchodilating mouse airway rings is shown in the figure legend. Compounds 111758, 87057, 3179204, 598689, and 2100101 inhibited the TMEM16A halide-sensitive YFP response with an average IC_{50} (µM) of 0.091±0.028 (n=3), 0.901±0.524 (n=53), 0.334 (n=1), 0.466±0.101 (n=3), and 3.330 (n=1), respectively.

3.36 Supplementary Figure 36

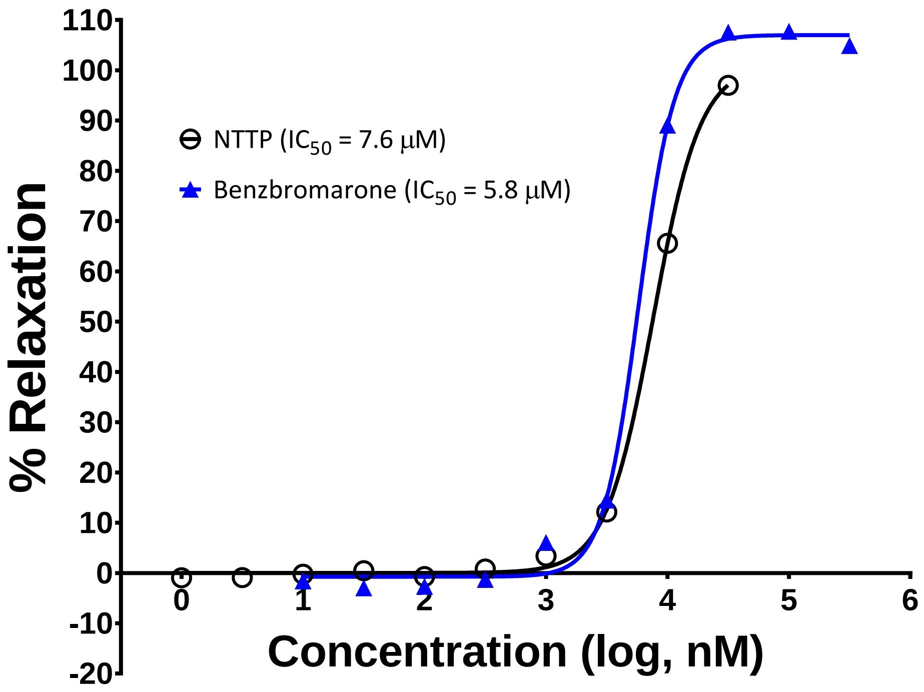

Supplementary Figure 36. **NTTP is efficacious in fully bronchodilating mouse tracheal rings.** The compound NTTP [CID: 19646] which only partially inhibited the TMEM16A *iodide* eYFP response but fully inhibited the calcium-activated *chloride* current (see Supplementary Figure 19), in physiological studies with tissue caused full relaxation of carbachol (EC_{75}) pre-contracted mouse tracheal rings.

3.37 Supplementary Figure 37

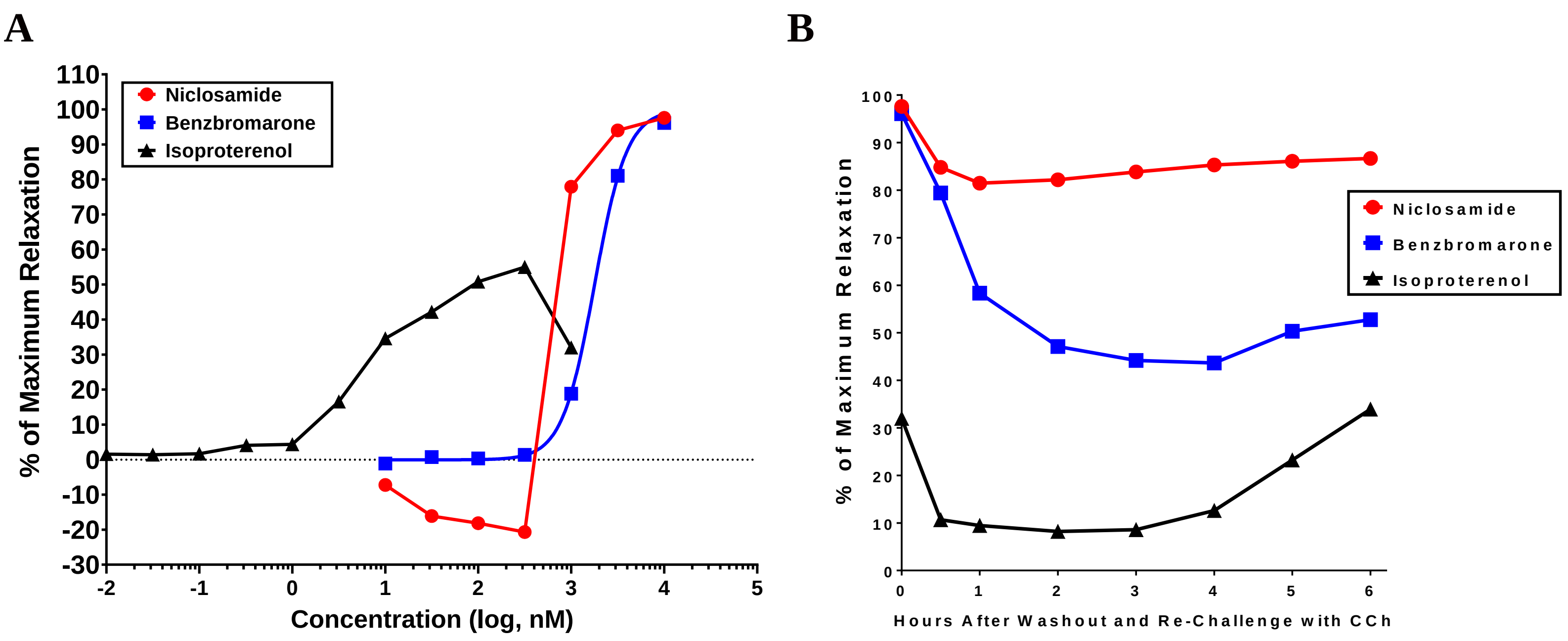

Supplementary Figure 37. TMEM16A antagonists fully relax airways and exhibit sustained action over several hours unlike the β-agonist isoproterenol which exhibited partial and transient bronchodilation. Panel **(A)** shows niclosamide, benzbromarone and isoproterenol caused dose-dependent relaxation of carbachol pre-contracted mouse tracheal rings. The airways were then washed to remove compounds and re-contracted with carbachol to evaluate if any of the compounds showed sustained effects **(B)**. The isoproterenol efficacy was quickly lost, but niclosamide and benzbromarone provided sustained bronchodilation lasting several hours.

3.38 Supplementary Figure 38

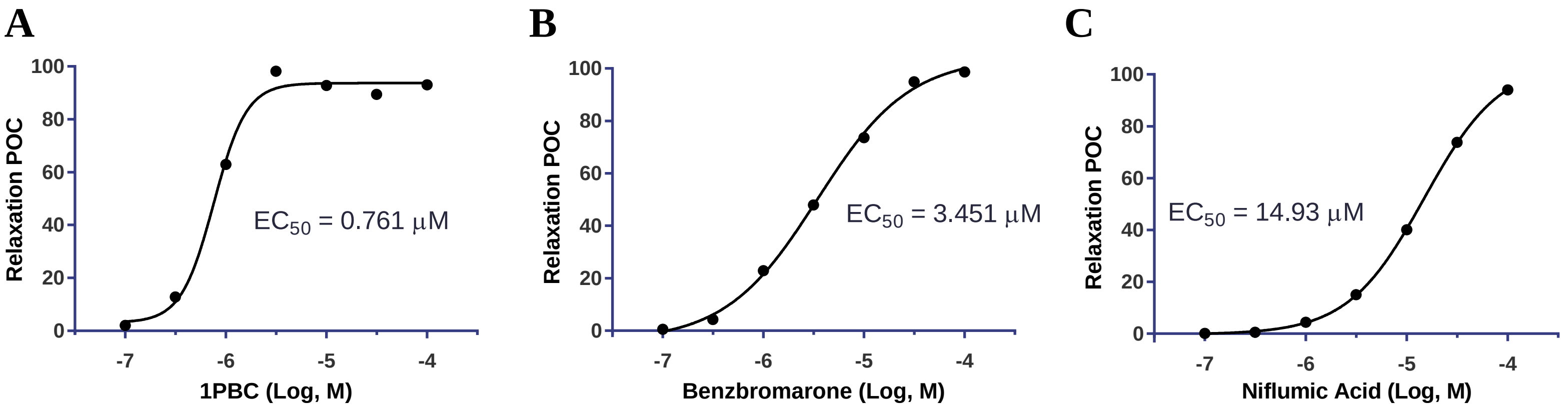

Supplementary Figure 38. **Amongst benchmark TMEM16A antagonists, 1PBC is routinely one of the best bronchodilators of human bronchial rings.** Cumulative concentration response curves (CCRC) for 1PBC **(A)**, benzbromarone **(B)** and niflumic acid **(C)** relaxation of airways pre-contracted with carbachol was measured using a DMT wire myograph, with the potency (EC_{50}) of each compound being listed. At the end of each recording and CCRC, theophylline was added to 2.2 mM final to induce maximal relaxation of the bronchial ring, which was considered 100 percent of control (POC) and used for normalization of compound effects. As a precaution, all recordings included 5 μM indomethacin in the bath solution to eliminate any possible indirect effect due to prostaglandins.

3.39 Supplementary Figure 39

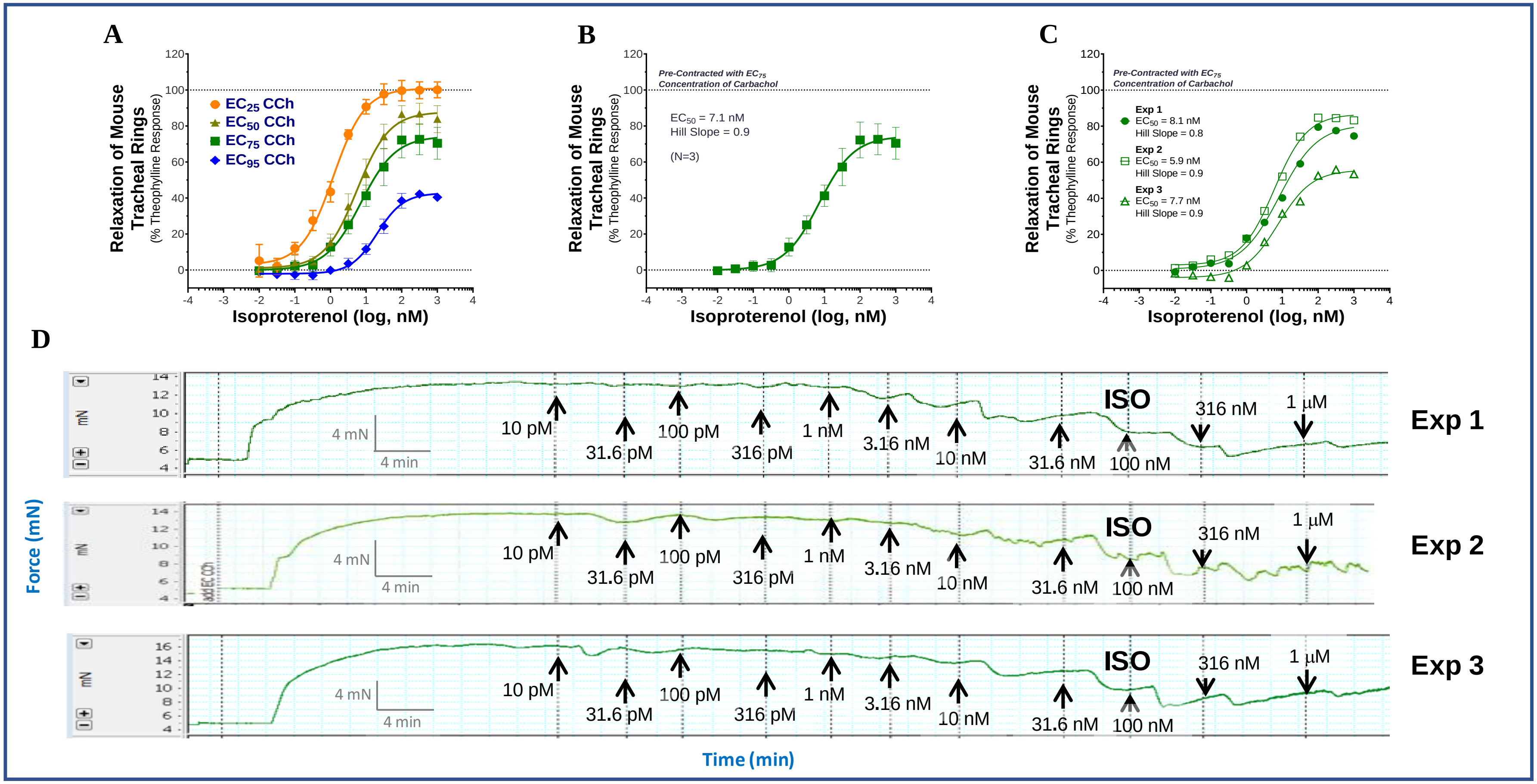

Supplementary Figure 39. **The β-agonist isoproterenol has a quick onset of action but does not fully relax maximally contracted mouse airways.** The potency and maximal efficacy of isoproterenol in bronchodilating mouse tracheal rings is progressively reduced with increasing concentrations of carbachol ranging from EC25 to EC95 **(A)**. The average efficacy **(B)**, individual airway dose-response results **(C)**, and raw wire myograph data of force changes over time **(D)** are shown for relaxation of airway rings pre-contracted with an EC75 concentration of carbachol. The hydrophilic compound isoproterenol (XLogP3 = -3.49) has a quick onset of action, taking just 2 – 4 min to relax the mouse tracheal tissue after applying a 10 nM dose **(D)**.

3.40 Supplementary Figure 40

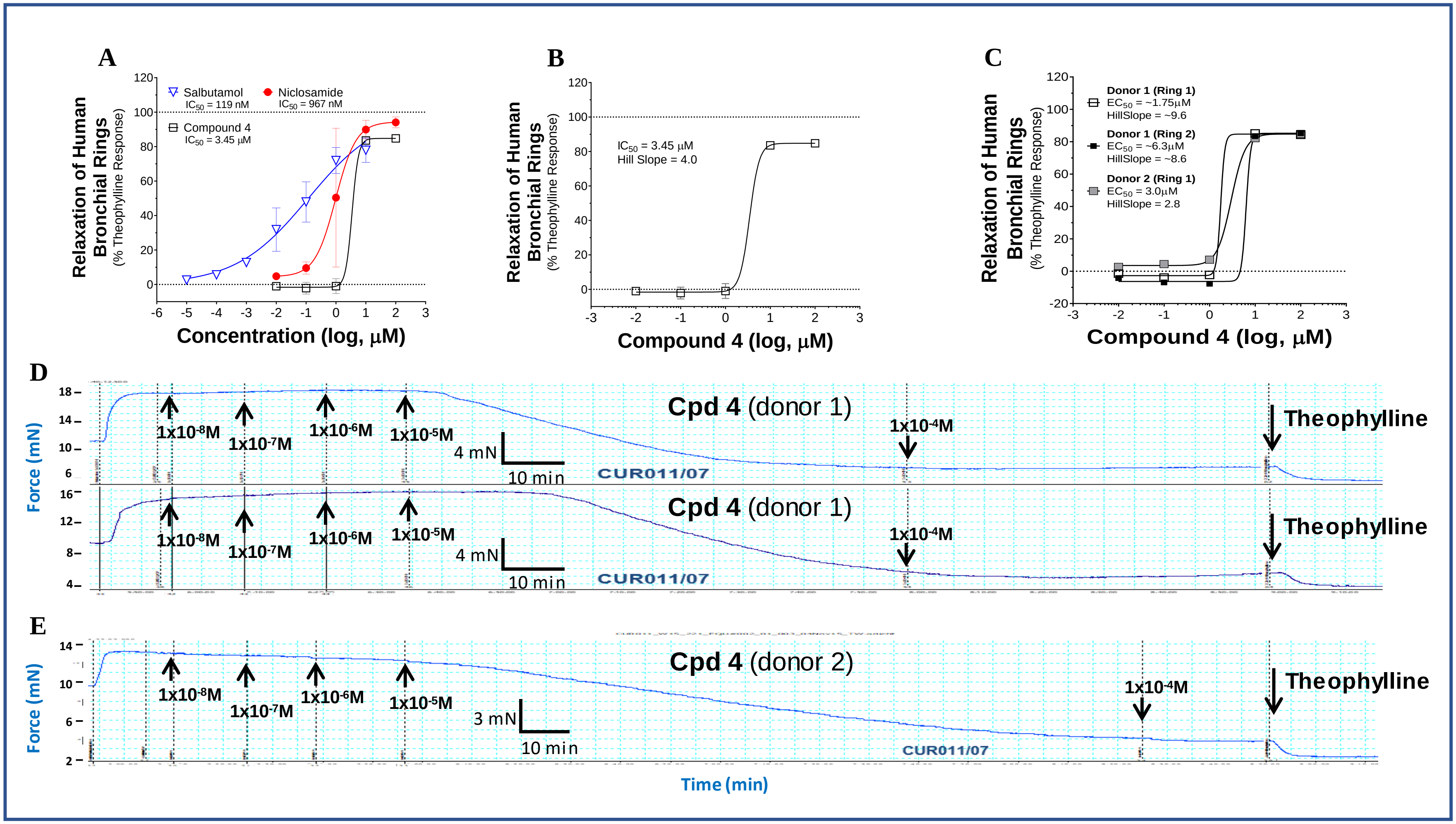

Supplementary Figure 40. The niclosamide-related compound, Cpd 4, fully relaxes human bronchial rings pre-contracted with histamine, but exhibits a slow onset of action. The relative efficacy of niclosamide and Compound 4 (Cpd 4) compared to the β-agonist salbutamol is shown in panel **(A)** for relaxation of human 4th order bronchi pre-contracted with histamine. The average efficacy **(B)**, individual airway dose-response results **(C)**, and raw wire myograph data of force changes over time **(D, E)** are shown for the lipophilic molecule Cpd 4 (XLogP3 = 4.63) which exhibits a slow onset of action taking between 50 – 125 min at 10 μM before there is steady-state relaxation of the human bronchial tissue **(D, E)**.

3.41 Supplementary Figure 41

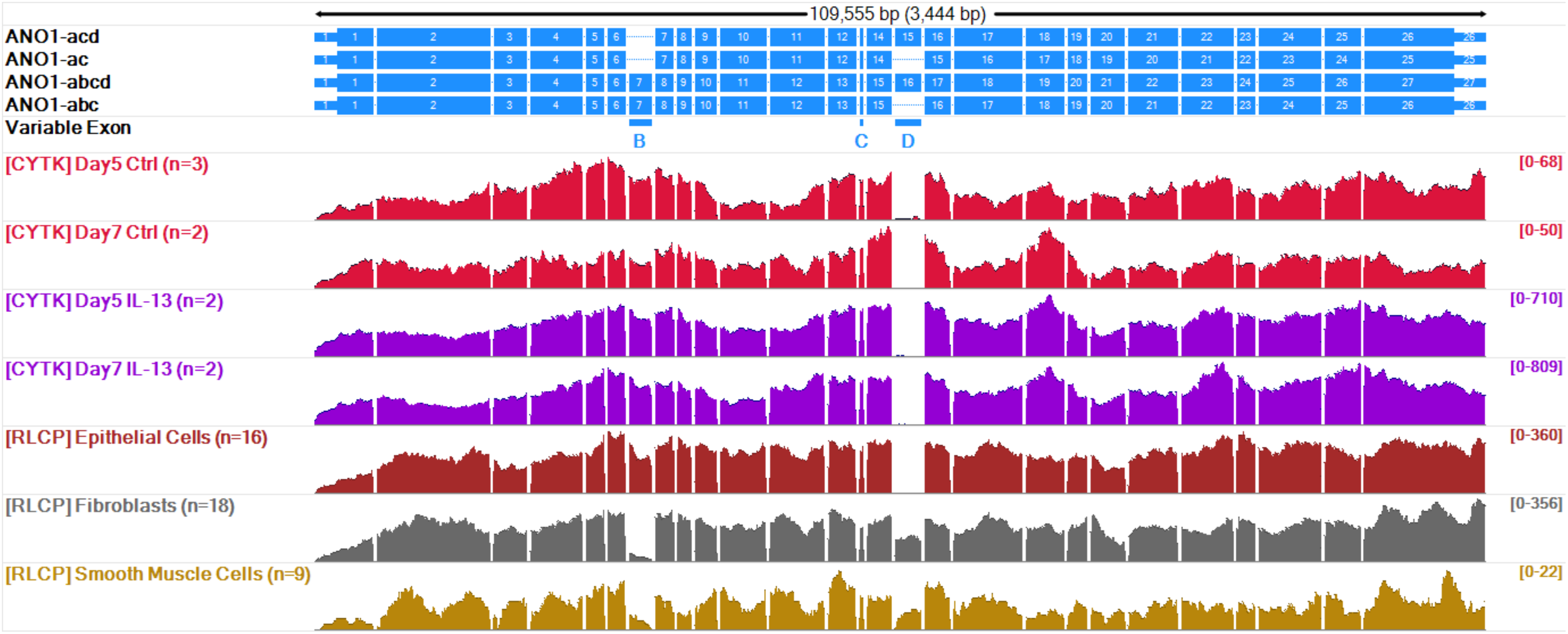

Supplementary Figure 41. **Array Studio visualization of RNA-Seq read coverage to validate expression and assess alternative splicing.** Submerged bronchial epithelial cells (RLCP Epithelial Cells) and fully differentiated bronchial epithelial cells (CYTK) from untreated (red; Day5-or Day7-Ctrl) or IL-13 treated (purple; Day5 or Day7) ALI cultures express predominantly the 'abc' splice variant of *TMEM16A* (*ANO1*). In contrast, cultured (RLCP) human airway smooth muscle cells (ocher) and fibroblasts (gray) show a mixture of splice variants, with segments A and C detected in nearly all, but variable expression of segments B and D. Since ASM cells express lower levels of *ANO1* than the bronchial epithelium, it is difficult to decipher the exact variants expressed in ASM cells. The track entitled 'Variable Exon' shows the location of segments B, C and D. The four transcripts (blue) diagrammed at the topic of the image represent the most common permutations of segment B, C, and D inclusion (segment A appears to be almost universally retained). Thick bars denote exon sequences, and thin bars denote UTR sequences, which have been truncated to emphasize coding exon coverage. Intronic regions common to all transcripts are omitted. The individual RNA-Seq coverage tracks show aggregate read coverage for samples from the Amgen Lung dataset (RLCP) described earlier (Aisenberg et al. 2016) and the ALI culture IL-13 dataset (CYTK) described in the Supplementary Methods. The scale on the right side of the track indicates the maximum coverage of any base within the region. This visualization was made using the Array Studio gene browser (Omicsoft, NC).

3.42 Supplementary Figure 42

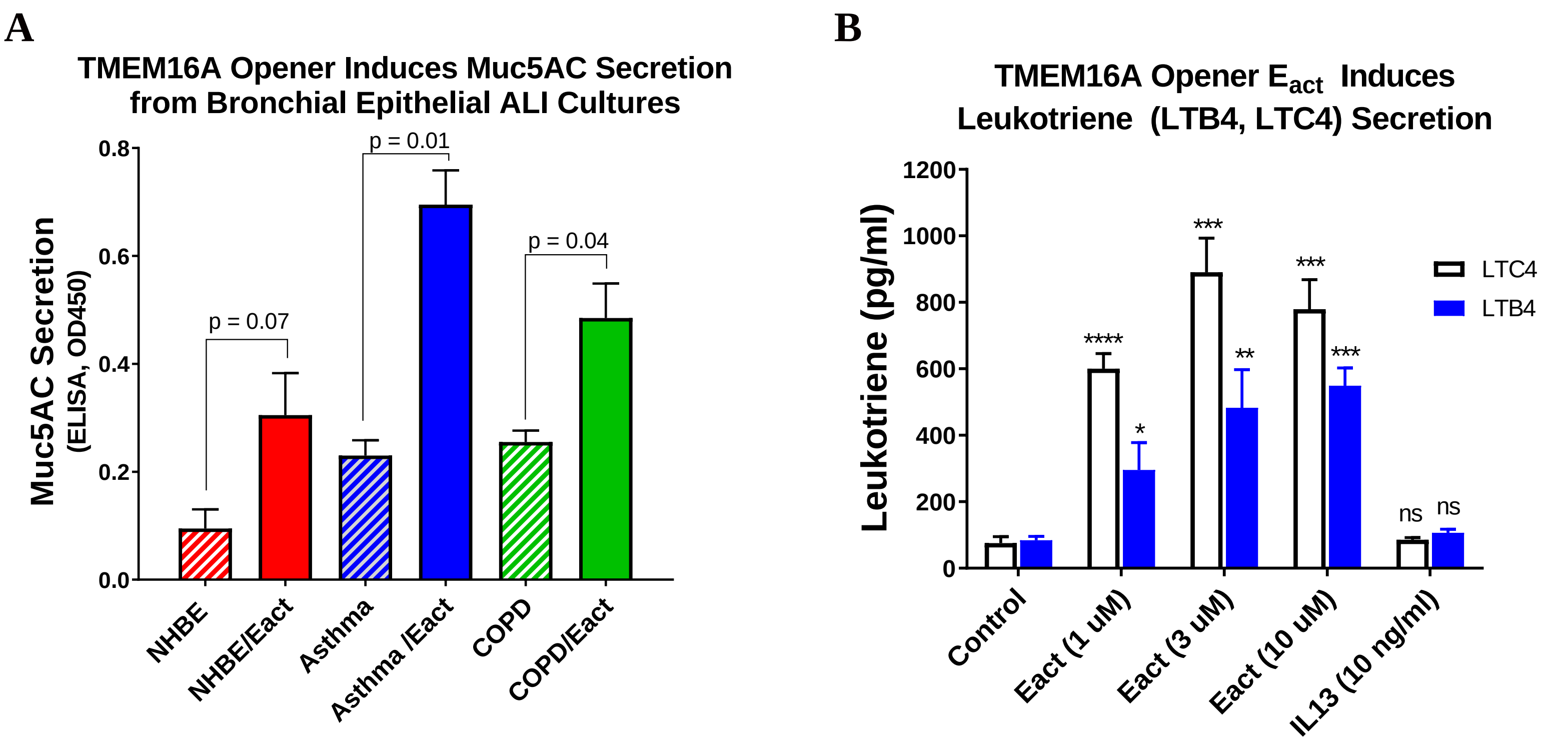

Supplementary Figure 42. **The TMEM16A opener E_{act} induces Muc5AC and leukotriene secretion from human bronchial epithelial (HBE) air-liquid interface (ALI) cultures.** Mature (14 day) HBE ALI cultures from normal, asthma or COPD patients (2 donors each, 2 ALI cultures per donor) were treated with vehicle or 5 µM Eact in basal medium for 72 hours and then the apical side of ALI cultures were washed with 10mM DTT at 37°C for 15min to collect mucus for MUC5AC ELISA analysis **(A)**. To measure effects on leukotriene secretion, normal HBE ALI cultures were treated with Eact vehicle (control), Eact opener, or 10ng/ml IL13 in basolateral medium for 48 hrs (n = 3 ALI cultures per condition), after which 100 µl of basolateral medium was collected and leukotriene B4 and C4 were determined by ELISA **(B)**. Shown is Mean ± SD. Unpaired students *t-test* to evaluate differences from untreated or control, with numeric P values listed or denoted as following: * $P<0.05$, ** $P<0.01$, ***$P<0.001$, **** $P<0.0001$, ns $P>0.05$

3.43 Supplementary Figure 43

Cell Line	Aliases	Tissue	Cancer	ANO1 FPKM
TE11	TE-11	Esophagus	Squamous Cell Carcinoma	396.1
MDAMB415	MDA-MB-415	Breast	Carcinoma	194.4
TT_ESOPHAGUS	T.T|T_T	Esophagus	Squamous Cell Carcinoma	162.8
SCC25	SCC-25	Tongue	Squamous Cell Carcinoma	132.7
EPLC272H	EPLC-272H	Lung	Squamous Cell Carcinoma	130.4
VMCUB1	VM-CUB1	Bladder	Transitional Cell Carcinoma	121.4
COLO201	COLO 201	Colon	Adenocarcinoma	120.5
PANC0403	Panc 04.03|Panc4.03	Pancreas	Adenocarcinoma	117.7
PATU8902	PA-TU-8902|PATU8902T	Pancreas	Pancreatic Ductal Adenocarcinoma	113.4
OE21		Esophagus	Squamous Cell Carcinoma	107.5
SW1417	SW-1417	Colon	Adenocarcinoma	103.2
LS123		Colon	Adenocarcinoma	101.6
ASPC1	AsPC-1	Pancreas	Pancreatic Ductal Adenocarcinoma	96.8
HS255T	Hs 255.T	Colon	Adenocarcinoma	96.1
CAPAN2	Capan-2	Pancreas	Pancreatic Ductal Adenocarcinoma	95.8
BHY		Head and Neck	Squamous Cell Carcinoma	94.7
MCAS		Ovary	Mucinous Cystadenocarcinoma	89.5
PANC0813	Panc 08.13|Panc8.13	Pancreas	Pancreatic Ductal Adenocarcinoma	88.9
COLO205	COLO 205	Colon	Adenocarcinoma	88.1
RERFLCAD2	RERF-LC-Ad2	Lung	Adenocarcinoma	88.0
HCC1954		Breast	Ductal Breast Carcinoma	85.9
SW480	SW-480	Colon	Adenocarcinoma	84.6
L33	L3.3	Pancreas	Carcinoma	83.3
PATU8988S	PA-TU-8988S	Pancreas	Pancreatic Ductal Adenocarcinoma	83.2
SNU324	NCI-SNU-324|SNU-324	Pancreas	Carcinoma	78.7
TE14	TE-14	Esophagus	Squamous Cell Carcinoma	75.7
5637		Bladder	Carcinoma	74.1
CORL23	COR-L23	Lung	Large Cell Carcinoma	72.3
SCC4	SCC-4	Tongue	Squamous Cell Carcinoma	72.3
BFTC905	BFTC-905	Bladder	Transitional Cell Carcinoma	72.1
T3M4	T3M-4	Pancreas	Pancreatic Ductal Adenocarcinoma	72.0
SNU899	NCI-SNU-899|SNU-899	Aerodigestive Tract	Squamous Cell Carcinoma	63.0
PECAPJ34CLONEC12	PE/CA-PJ34 (clone C12)	Oral Cavity	Squamous Cell Carcinoma	62.7
SNU1197	NCI-SNU-1097|SNU-1197	Large Intestine	Carcinoma	61.4
SW1990	SW 1990|SW-1990	Pancreas	Pancreatic Ductal Adenocarcinoma	58.0
BICR22	BICR 22	Tongue	Squamous Cell Carcinoma	56.6
SNU1196	NCI-SNU-1196|SNU-1196	Biliary Tract	Carcinoma	55.2
BICR31	BICR 31	Tongue	Squamous Cell Carcinoma	54.3
PANC0203	Panc 02.03|Panc2.03	Pancreas	Adenocarcinoma	52.7
HPAC		Pancreas	Pancreatic Ductal Adenocarcinoma	52.2
CAL148	CAL-148	Breast	Ductal Breast Carcinoma	52.0
CAL12T	CAL-12T	Lung	Non-Small Cell Carcinoma	51.4
PK59	PK-59	Pancreas	Carcinoma	49.2
PC3	PC-3	Prostate Gland	Adenocarcinoma	49.1
YAPC		Pancreas	Pancreatic Ductal Adenocarcinoma	47.9
HCC1428		Breast	Adenocarcinoma	47.4
SNU81	NCI-SNU-81|SNU-81	Large Intestine	Carcinoma	46.6
DANG	DAN-G	Pancreas	Carcinoma	46.5
A253	A-253	Submandibular Gland	Mucoepidermoid Carcinoma	45.9
SNU719	NCI-SNU-719|SNU-719	Stomach	Carcinoma	45.1
UACC893	UACC-893	Breast	Ductal Breast Carcinoma	44.5
HCC2218		Breast	Ductal Breast Carcinoma	44.4
PANC0327	Panc 03.27|Panc3.27	Pancreas	Pancreatic Ductal Adenocarcinoma	43.5
DV90	DV-90	Lung	Adenocarcinoma	43.5
HCC1500		Breast	Ductal Breast Carcinoma	43.1

Cell Line	Aliases	Tissue	Cancer	ANO1 FPKM
CL11	CL-11	Colon	Adenocarcinoma	42.1
SKCO1	SK-CO-1	Colon	Adenocarcinoma	42.0
BC3C	BC-3C	Bladder	Transitional Cell Carcinoma	41.5
TE9	TE-9	Esophagus	Squamous Cell Carcinoma	41.2
CJM		Skin	Melanoma	40.4
HPAFII	HPAF-II	Pancreas	Pancreatic Ductal Adenocarcinoma	40.4
HUG1N	HuG1-N	Stomach	Carcinoma	39.7
NCIH854	H854|NCI-H854	Lung	Adenocarcinoma	38.1
RCM1	RCM-1	Rectum	Adenocarcinoma	38.1
MDAMB134VI	MDA-MB-134|MDA-MB-134-VI	Breast	Ductal Breast Carcinoma	38.0
OCUM1	OCUM-1	Stomach	Adenocarcinoma	37.9
CAPAN1	Capan-1	Pancreas	Pancreatic Ductal Adenocarcinoma	37.8
HCC1419		Breast	Ductal Breast Carcinoma	35.4
HCT116	HCT 116|HCT-116	Colon	Carcinoma	35.3
PK1	PK-1	Pancreas	Carcinoma	35.3
OE19		Lower Third of the Esophagus	Adenocarcinoma	34.8
YD38	YD-38	Aerodigestive Tract	Squamous Cell Carcinoma	34.3
SNU620	NCI-SNU-620|SNU-620	Stomach	Carcinoma	33.6
SNUC5	SNU-C5	Large Intestine	Adenocarcinoma	31.1
LU65		Lung	Non-Small Cell Carcinoma	31.1
MDAMB175VII	MDA-MB-175|MDA-MB-175-VII	Breast	Ductal Breast Carcinoma	31.0
OE33		Lower Third of the Esophagus	Adenocarcinoma	30.9
SCC15	SCC-15	Tongue	Squamous Cell Carcinoma	30.8
SNU175	NCI-SNU-175|SNU-175	Large Intestine	Carcinoma	30.3
ZR751	ZR-75-1	Breast	Ductal Breast Carcinoma	29.9
HUPT3	HUP-T3	Pancreas	Pancreatic Ductal Adenocarcinoma	29.5
LS180	LS 180	Colon	Adenocarcinoma	29.0
CAL27	CAL 27	Tongue	Squamous Cell Carcinoma	28.9
BICR18	BICR 18	Larynx	Squamous Cell Carcinoma	28.8
JHOM2B	JHOM-2B	Ovary	Mucinous Adenocarcinoma	28.7
PC14	PC-14	Lung	Non-Small Cell Carcinoma	28.5
PANC0504	Panc 05.04	Pancreas	Adenocarcinoma	27.9
PECAPJ15	PE/CA-PJ15	Tongue	Squamous Cell Carcinoma	27.8
SW1116	SW 1116|SW-1116	Colon	Adenocarcinoma	27.8
JHUEM3	JHUEM-3	Uterus	Adenocarcinoma	26.9
BT474	BT-474	Breast	Ductal Breast Carcinoma	26.5
HT29	HT-29	Colon	Adenocarcinoma	26.7
KP2	KP-2	Pancreas	Tubular Adenocarcinoma	26.4
RERFLCAD1	RERF-LC-Ad1	Lung	Adenocarcinoma	26.3
DU4475		Breast	Ductal Breast Carcinoma	25.8
NCIH1648	H1648|NCI-H1648	Lung	Adenocarcinoma	25.7
IM95		Stomach	Adenocarcinoma	25.4
CFPAC1	CFPAC-1	Pancreas	Pancreatic Ductal Adenocarcinoma	25.1
SW837	SW-837	Rectum	Adenocarcinoma	23.7
KYSE140	KYSE-140	Esophagus	Squamous Cell Carcinoma	23.4
SNU1076	NCI-SNU-1076|SNU-1076	Aerodigestive Tract	Squamous Cell Carcinoma	23.3
RERFLCKJ	RERF-LC-KJ	Lung	Non-Small Cell Carcinoma	23.1
HS695T	Hs 695T	Skin	Amelanotic Melanoma	22.7
NUGC3	NUGC-3	Stomach	Adenocarcinoma	21.8
UMUC1	UM-UC-1	Bladder	Transitional Cell Carcinoma	21.5
HUCCT1	HuCCT1	Bile Duct	Carcinoma	20.5
TE15		Esophagus	Squamous Cell Carcinoma	20.1
NCIH2347	H2347|NCI-H2347	Lung	Adenocarcinoma	20.1
SNU407	NCI-SNU-407|SNU-407	Large Intestine	Carcinoma	20.0
LS411N		Cecum	Adenocarcinoma	20.0

Supplementary Figure 43. ***TMEM16A* (*ANO1*) expression in cancer cell lines.** The RNA-Seq *ANO1* expression level is reported for cell lines from the Cancer Cell Line Encyclopedia (Barretina et al. 2012), one cell line present in both CCLE and Amgen (HT-29; average FPKM shown) and a single cell lines assayed at Amgen, COLO205. The results for cancer cell lines exhibiting the highest expression (FPKM ≥ 20) are shown, and in all cases except HT-29 sample size is one.

3.44 Supplementary Figure 44

A

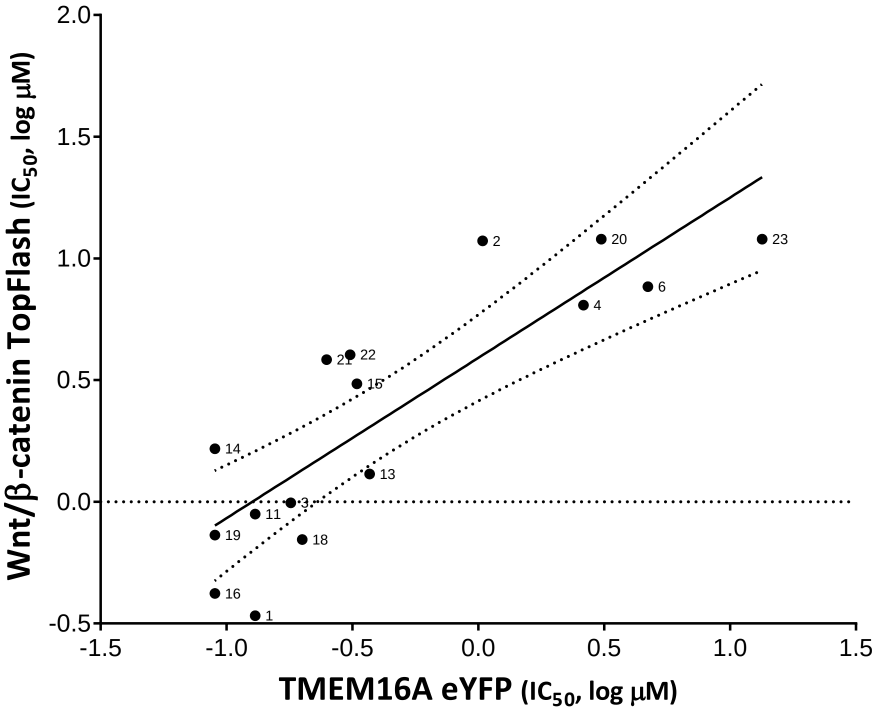

B

Compound No. (RM#)	TMEM16A eYFP Assay (IC50, μM)	Mook et al. Wnt/β-catenin TopFlash (IC50, μM)	IC50 Ratio, Wnt/TMEM16A
1	0.13	0.34	2.62
2	1.04	11.81	11.36
3	0.20	0.99	5.50
4	2.62	6.42	2.45
6	4.72	7.66	1.62
11	0.13	0.89	6.85
13	0.37	1.30	3.51
14	0.09	1.65	18.33
15	0.33	3.05	9.24
16	0.09	0.42	4.67
18	0.20	0.70	3.50
19	0.09	0.73	8.11
20	3.08	>12	NA
21	0.25	3.84	15.36
22	0.31	4.02	12.97
23	13.4	>12	NA

Supplementary Figure 44. Activity of sixteen niclosamide compounds on TMEM16A versus Wnt/β-catenin. Mook et al. (2015) describe the structure and activity of various niclosamide analogs for inhibition of Wnt/β-catenin transcription using a TopFlash assay. Sixteen of these compounds we have also tested for activity in our halide-sensitive TMEM16A eYFP assay and the activity is compared in the plot provided in panel **(A)**. Pearson's analysis of correlation (n=14) using GraphPad Prism 7.03, provided an r of 0.8046 and P (two-tailed) of 0.0005. The raw IC_{50} values from each compound are provided in the table **(B)**, where compound numbering is as provided by Mook et al. (2015), with Compound No. 1 being niclosamide.

4 Supplementary References

Aisenberg, W. H., J. Huang, W. Zhu, P. Rajkumar, R. Cruz, L. Santhanam, N. Natarajan, H. M. Yong, B. De Santiago, J. J. Oh, A. R. Yoon, R. A. Panettieri, O. Homann, J. K. Sullivan, S. B. Liggett, J. L. Pluznick, and S. S. An. 2016. "Defining an olfactory receptor function in airway smooth muscle cells." *Sci Rep* 6:38231. doi: 10.1038/srep38231.

Barretina, J., G. Caponigro, N. Stransky, K. Venkatesan, A. A. Margolin, S. Kim, C. J. Wilson, J. Lehar, G. V. Kryukov, D. Sonkin, A. Reddy, M. Liu, L. Murray, M. F. Berger, J. E. Monahan, P. Morais, J. Meltzer, A. Korejwa, J. Jane-Valbuena, F. A. Mapa, J. Thibault, E. Bric-Furlong, P. Raman, A. Shipway, I. H. Engels, J. Cheng, G. K. Yu, J. Yu, P. Aspesi, Jr., M. de Silva, K. Jagtap, M. D. Jones, L. Wang, C. Hatton, E. Palescandolo, S. Gupta, S. Mahan, C. Sougnez, R. C. Onofrio, T. Liefeld, L. MacConaill, W. Winckler, M. Reich, N. Li, J. P. Mesirov, S. B. Gabriel, G. Getz, K. Ardlie, V. Chan, V. E. Myer, B. L. Weber, J. Porter, M. Warmuth, P. Finan, J. L. Harris, M. Meyerson, T. R. Golub, M. P. Morrissey, W. R. Sellers, R. Schlegel, and L. A. Garraway. 2012. "The Cancer Cell Line Encyclopedia enables predictive modelling of anticancer drug sensitivity." *Nature* 483 (7391):603-7. doi: 10.1038/nature11003.

Mook, R. A., Jr., J. Wang, X. R. Ren, M. Chen, I. Spasojevic, L. S. Barak, H. K. Lyerly, and W. Chen. 2015. "Structure-activity studies of Wnt/beta-catenin inhibition in the Niclosamide chemotype: Identification of derivatives with improved drug exposure." *Bioorg Med Chem* 23 (17):5829-38. doi: 10.1016/j.bmc.2015.07.001.

Namkung, W., Z. Yao, W. E. Finkbeiner, and A. S. Verkman. 2011. "Small-molecule activators of TMEM16A, a calcium-activated chloride channel, stimulate epithelial chloride secretion and intestinal contraction." *FASEB J* 25 (11):4048-62. doi: 10.1096/fj.11-191627.

Wang, Y. X., and M. I. Kotlikoff. 1997. "Inactivation of calcium-activated chloride channels in smooth muscle by calcium/calmodulin-dependent protein kinase." *Proc Natl Acad Sci U S A* 94 (26):14918-23.
